# Characterization and Comparison of Dietary Taste Patterns in Five Countries With Differing Obesity Prevalence

**DOI:** 10.1002/fsn3.72307

**Published:** 2026-09-16

**Authors:** Claudia S. Tang, Gilly A. Hendrie, David N. Cox, Monica Mars, Kees de Graaf, Katherine M. Appleton

**Affiliations:** ^1^ Department of Psychology, Faculty of Science and Technology Bournemouth University Poole Dorset UK; ^2^ Health and Biosecurity, Commonwealth Scientific and Industrial Research Organisation (CSIRO) Adelaide South Australia Australia; ^3^ Sensory Science and Eating Behaviour Group, Division of Human Nutrition Wageningen University and Research Wageningen Gelderland the Netherlands

**Keywords:** agglomerative hierarchical cluster analysis, dietary intake, eating rate, oro‐sensory exposure, sensory database

## Abstract

Considering associations between taste, food intake, and overconsumption, this work aimed to characterize and compare dietary taste patterns across five countries with differing rates of obesity prevalence: France (10% of the population), the Netherlands (15%), the United Kingdom (UK) (27%), Australia (32%), and the United States (US) (44%). Taste intensity values for all five basic tastes and fatty mouthfeel were assigned to all foods in national dietary intake records. Dietary taste patterns were then characterized and compared using taste clusters and taste exposure. In cluster analyses, all five diets included “neutral”, “bitter” and “sweet” taste clusters, with differences in the other taste clusters present. For taste exposure, sweet taste exposure was highest in all diets and contributed most to total exposure, salt taste and fatty mouthfeel exposure were also high. Salt taste and umami taste contributed most to the US diet, while bitter taste was highest in the Dutch diet, and sour taste was highest in the French diet. Similarities across countries point to the dominance of sweet taste in consumption. Differences between countries provide some suggestions that salt and umami tastes contribute more in countries with higher obesity prevalence, and that bitter and sour tastes contribute more where obesity prevalence is lower.

## Graphical abstract

This work investigated dietary taste patterns in five countries with differing rates of obesity prevalence: France (10% of the population), the Netherlands (15%), the United Kingdom (27%), Australia (32%), and the United States (44%). Methods were trialed first, then countries were compared. All countries were found to consume diets high in sweet and neutral tastes, while salt taste and umami taste contributed most to the American diet, bitter taste was highest in the Dutch diet, and sour taste was highest in the French diet.

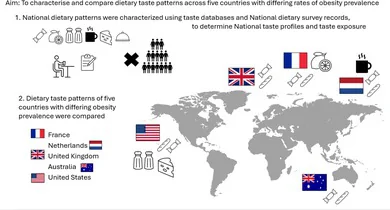

## 1 Introduction

Obesity prevalence is increasing globally (NCD Risk Factor Collaboration (NCD‐RisC) 2024), driven, at least in part, by increased consumption. Taste plays a pivotal role in consumption, such that it has been postulated that the hedonic value of certain taste modalities, such as sweet and salty, promotes the consumption of energy‐dense foods (Spinelli and Monteleone 2021). Tastes are associated with some nutrients (Lease et al. 2016; Martin and Issanchou 2019; Teo et al. 2022, 2018; van Langeveld et al. 2017), and dietary taste patterns are related to nutrient intakes (van Langeveld et al. 2018). Taste is also associated with acceptance and liking (Bertino et al. 1986; Kourouniotis et al. 2016), can activate reward signaling in the brain (Rolls 2015), and influence satiation and satiety (Low et al. 2014; McCrickerd and Forde 2016). All these factors are associated with food choice and intake (van Langeveld et al. 2018; Bertino et al. 1986; Kourouniotis et al. 2016; Rolls 2015; Low et al. 2014; McCrickerd and Forde 2016), and for this reason, an increased understanding of dietary intake from a taste perspective may be of value.

Obesity rates differ greatly between countries, perhaps partially, due to differing dietary patterns (Bliznashka et al. 2021; Swarnamali et al. 2022). To date, the taste profiles of dietary patterns have been explored in nationally representative diets in Australia, Singapore, Malaysia, and the Netherlands (Teo et al. 2022; van Langeveld et al. 2018; Cox, Hendrie, Lease, Rebuli, and Barnes 2018). The sweetness, saltiness, sourness, savouriness, bitterness, and fatty mouthfeel of the diets from each country were quantified using taste databases to provide a national dietary taste profile. However, methodological differences in quantifying dietary taste currently prevent comparisons between countries.

Current databases contain taste intensity values for a range of foods, as profiled by trained sensory panelists (Lease et al. 2016; Teo et al. 2022; Mars et al. 2020; Martin et al. 2014), but the number of foods for which data are available is limited by the time and resources required. In order to examine population dietary taste profiles, however, taste intensity values are required for all consumed foods. Missing taste values have previously been assigned using a “best match” approach based on sensory characteristics (Cox, Hendrie, Lease, Rebuli, and Barnes 2018) or using a measure of central tendency, such as the average intensity values of tested foods within a food (sub‐)group (Teo et al. 2022, 2018; van Langeveld et al. 2018).

Further to these methodological differences, the contribution of each taste from all recorded foods to create dietary patterns has also been determined in different ways. In characterizing dietary taste profiles for Singapore, Malaysia and the Netherlands, researchers applied agglomerative hierarchical clustering (Teo et al. 2022, 2018; van Langeveld et al. 2018), while the dietary taste profile of Australia was characterized by calculating “taste amount” and “taste density”, an attribute based on the proportion of taste to energy content (Cox, Hendrie, Lease, Rebuli, and Barnes 2018).

The above‐mentioned studies also focused only on the quality (e.g., sweet, sour) and intensity aspects of taste. The magnitude of taste experienced during consumption, however, is also determined by the duration of exposure (Lasschuijt et al. 2021), which is largely determined by oral processing and eating rate (de Graaf 2012). Importantly, taste exposure, considering both taste intensity and duration of exposure, has been found to affect both the quantity and quality of foods consumed (see review: (Stribiţcaia et al. 2020)).

Considering the importance of taste in food intake and the associations between food intake and obesity, there is a need to better understand dietary intake from a taste perspective. While food intake and obesity vary by population, value may be gained from comparing population groups. The primary aim of this work was to characterize and compare dietary taste patterns across several countries, dependent on obesity prevalence. Countries were selected considering obesity prevalence, the availability of national dietary consumption records and taste databases, resulting in the selection of France (10% of the population reported as living with obesity), the Netherlands (15%), the United Kingdom (UK) (27%), Australia (32%), and the United States (US) (44%) (NCD Risk Factor Collaboration (NCD‐RisC) 2024). In advance of this primary aim, we aimed first to investigate and determine the most suitable method for characterizing the dietary taste profile for each country in a manner that would fully describe each diet and allow cross‐country comparison (methodological aim).

## 2 Methods

### 2.1 Methodological Aim: To Investigate and Determine the Most Suitable Method for Characterizing the Dietary Taste Profile of a Country in a Manner That Would Allow Full Description and Cross‐Country Comparison

To characterize the dietary taste profile for each country, we first needed to assign taste values to all foods in national dietary records, including those not included in taste databases. Second, a method for comparing countries was needed. Various approaches were applied to the national diet of Australia and evaluated for validity, suitability, and feasibility.

#### 2.1.1 Dietary Records

The National Nutrition and Physical Activity Survey (NNPAS) of Australia 2011–2012 was used to typify the national diet of Australia (Australian Bureau of Statistics 2013). Participants of this survey were 6415 adults (3305 female, mean age, 42.0 ± 13.1 years, mean BMI, 27.3 ± 5.6 kg/m^2^) who provided data in two nonconsecutive 24 h dietary recalls completed by trained dietitians. A total of 5648 unique food codes were reported; each food belonging to a major, sub‐major, and minor food group according to the Australian Food, Supplement and Nutrient Database (AUSNUT) (Australian Bureau of Statsitics 2023).

#### 2.1.2 Taste Values

All foods consumed in the NNPAS were then assigned taste values describing the intensity of five basic tastes—sweet, sour, salt, bitter, umami, and fatty mouthfeel. Throughout this work, we focus on “fatty mouthfeel” rather than “fat taste” (Keast et al. 2021) as a reflection of the taste databases used (Lease et al. 2016; Mars et al. 2020; Martin et al. 2014). Where possible, taste intensity values were taken from the Australian Sensory‐Diet Database (Lease et al. 2016), a database comprising the taste profiles of 720 frequently consumed foods representative of the Australian diet. Taste values for alcoholic beverages, sweetened coffee, and sweetened tea (*n* = 22) are not included in the Sensory‐Diet Database, so were taken instead from the Dutch Taste, Fat and Texture Database (Mars et al. 2020), assuming these foods taste similar across countries.

For other foods not present in the Australian Sensory‐Diet Database (*n* = 4906), taste values were imputed using three methods:

(1) “Best match”: Each untested food was matched with a tested food based on similarities in food description and nutrient content by individuals with expertise in food composition and sensory profiling from Australia with knowledge of the Australian food supply, as previously described (Lease et al. 2016).

(2) “Group mean”: All untested foods within a food (sub‐)group were assigned the mean values of all tested foods within an AUSNUT (sub‐)group.

(3) “Group median”: All untested foods within a food (sub‐)group were assigned the median values of all tested foods within an AUSNUT (sub‐)group.

Paired sample *t*‐tests and Bland–Altman plots were used to statistically evaluate agreement between “best match” and the other two methods, with the “best match” method as the reference, since it allows item‐specificity by experts.

Following imputation, average taste and mouthfeel values differed significantly depending on the imputation method. However, differences were smaller between the reference and “group mean” (range: 0.15–1.19; smallest *t*(5647) = 3.43, *p* < 0.001), than between the reference and “group median” (range: 0.11–2.17; smallest *t*(5647) = 8.35, *p* < 0.001). Only the assigned values for salt taste and sour taste did not differ between the reference and “group mean” (*t*(5647) = −1.32, *p* = 0.19; *t*(5647) = −1.34, *p* = 0.18, respectively). Both “group mean” and “group median” displayed similar patterns on Bland–Altman plots compared to the reference, but the larger the intensity value, the larger the difference. Average taste and mouthfeel intensity values for all foods, and all results of the different imputation methods are provided in Table S1.1, Table S1.2, and Figure S1.1.

For evaluation, all three methods faced the same limitation that foods within a food (sub‐) group may not taste the same, since food (sub‐) groups in food composition databases are typically formed based on nutrient composition rather than taste. For example, pickled, smoked, and salted cabbage all belong to the food sub‐group vegetables, but taste very different. The “best match” method allowed item‐specificity, but required native experts from the country in sensory fields to match each untested food to a tested food, so it was time‐consuming and expensive, and if none or only one food was tested in a food sub‐group, it offered no advantage over taking a measure of central tendency. Measures of central tendency, by comparison, could be applied by a non‐native, and between the two measures, “group mean” showed smaller differences from “best match” than “group median”. Strengths (+) and limitations (−) of each method for assigning taste values are given in Table 1 (panel A). Considering these strengths and limitations, the “group mean” method was considered most appropriate for imputing missing taste values.

**TABLE 1:** Strengths (+) and limitations (−) of each method for (A) Imputing missing taste values and (B) Characterizing dietary taste patterns.

(A) Assigning taste values		
Best match (reference)	Group mean	Group median
+ Item‐specificity and variation	+ Objective	+ Objective
+ Expert evaluation	+ Less time‐consuming	+ Less time‐consuming
– Time‐consuming and expensive	– Foods within a sub‐group may not taste the same	– Foods within a sub‐group may not taste the same
– Foods within a sub‐group may not taste the same	– Systematically assigns higher values	– Systematically assigns higher values
− No benefit/advantage over other methods if ≤ 1 food tested in a sub‐group		

(B) Characterizing dietary taste patterns		
Cluster (reference)	Taste amount	Taste exposure
+ Dominant taste combinations + Allows consideration of neutral foods – Dataset specific – Comparison requires cluster formation on all databases together	+ Intensity of taste exposed during eating + Absolute values and patterns of dietary taste + Regardless of energy density + Use of the six basic tastes/mouthfeel allow comparison between groups – Does not allow for neutral (non‐tasting) foods – Duration of taste exposed not captured	+ Intensity and duration of taste exposed during eating + Absolute values and patterns of dietary taste + Regardless of energy density + Use of the six basic tastes/mouthfeel allow comparison between groups – Does not allow for neutral (non‐tasting) foods – Estimation of eating durations

#### 2.1.3 Dietary Characterization

With all foods in the NNPAS assigned taste and mouthfeel intensity values, three methods to characterize each diet, per participant, were then investigated:

(1) “Cluster”: All foods were grouped according to similarity in taste values using agglomerative hierarchical cluster analysis (using cophenetic correlation coefficient (c) and Wilks' lambda (λ), with Ward's clustering and Euclidean distance measures) (see Tables S2.1–S2.5 and Figure S2.1, for details). Taste clusters were data‐driven, where each cluster ideally had a unique dominant taste or taste combination with a minimum number of qualifiers, where the foods included were also logical. Subsequently, quantity consumed (in grams) from each taste cluster was summed per dietary recall day, averaged per person, and calculated as a percentage of total intake. Weight of food, rather than energy, consumed, allowed for consideration of foods that contribute weight and taste to the diet, but do not contribute energy, for example, low‐calorie sweetened beverages.

(2) “Taste amount” (score g): For this method, taste intensity values (scores) for all tastes and for fatty mouthfeel for each food were multiplied by the quantity of each food consumed (g). These were then averaged across dietary recall days to obtain a mean daily taste amount (score g) for each taste, per participant.

(3) “Taste exposure” (score min): This method stemmed from “taste amount”, while also considering the sensory impact during consumption based on time to consume. Taste amount (score g) was multiplied by a constant representing the average eating duration for 100 g food, based on its form. Food form (liquid, semi solid, soft solid, hard solid) was decided based on previous definitions (see Table S3.1) (Stieger and van de Velde 2013). Constants were based on the Dutch Eating Rate Database (van den Boer et al. 2017) as time required to consume 100 g of liquid: 0.33 min, semi‐solid: 1.59 min, soft‐solid: 3.33 min, hard‐solid: 5.26 min. Taste exposure (score min) was then summed, per taste, per day, and averaged across all recall days to obtain a mean daily taste exposure (score min) per taste per participant.

Table 2 illustrates the differences between taste intensity values, taste amount, and taste exposure for sweet taste in four chocolate products with different sensory impacts due to differences in food form; “taste amount” reflects amount consumed, whereas “taste exposure” reflects amount consumed and time in the mouth, where because solid foods take longer to consume than liquid products, taste exposure is greater.

**TABLE 2:** Examples of sweet taste intensity, amount, and exposure across four chocolate items differing in food form.

	Milk chocolate bar	Milk chocolate bar	Chocolate mousse	Chocolate custard	Chocolate milk	Chocolate milk
Food form	Solid	Solid	Soft‐solid	Semi‐solid	Liquid	Liquid
Consumed quantity (grams)	100	50	100	100	100	50
Sweet taste intensity (score)*	55	55	46	34	37	37
Sweet taste amount (score g)	5500	2750	4600	3400	3700	1850
Eating duration constant (min/100 g)**	5.26	5.26	3.33	1.59	0.33	0.33
Sweet taste exposure (score min)	28,930	14,465	15,318	5406	1221	621

* Obtained from the Dutch taste database (Mars et al. 2020).

** Obtained from the Dutch Eating Rate database (van den Boer et al. 2017).

#### 2.1.4 Dietary Comparison: Australia

To evaluate these methods to characterize and compare dietary taste profiles, the diets of male and female Australian adults were selected to investigate methods for comparison. These two groups were selected simply to allow investigation of several methods for comparison—we had no a prior interest in investigating differences between genders.

##### 2.1.4.1 Cluster

Using agglomerative hierarchical cluster analysis, six clusters emerged for both male and female datasets. These clusters were labeled “Neutral”, “Bitter”, “Sweet”, “Sweet Sour”, “Salt Fat”, and “Fat” based on the taste of the foods in the cluster that was most prominent. For both males and females, the highest contribution to total intake came from neutral‐tasting foods (males 42%, females 49%), followed by contributions from foods tasting bitter (males 26%, females 19%), sweet (males 12.5%, females 12%), sweet sour (males 12%, females 11.5%), salt fat (males 11.5%, females 11%), and fat (males 8.5%, females 8%) (see Figure S3.1).

##### 2.1.4.2 Taste Amount

Total taste amount (all taste modalities together) was greater for males (mean: 201,110 score g) than for females (mean: 155,254 score g) (see Figure S3.2). For both sexes, the contributions of each taste to total taste amount were similar. Sweet taste amount contributed most (males/females, 27%), followed by the taste amount from bitter (males 24%; females 23%), salt (males 15%; females 16%), fatty mouthfeel (males 14%; females 15%), and sour (males/females 14%), while umami taste amount was lowest (males/females 6%).

##### 2.1.4.3 Taste Exposure

Total taste exposure was also greater for males (mean: 364,098 score min) than for females (mean: 300,179 score min) (see Figure S3.3). For both sexes, the contributions of each taste to total taste exposure were similar. Sweet taste exposure contributed most (males 25%; females 24%), followed by exposure to salt taste (males/females 21%), fatty mouthfeel (males/females 19%), bitter taste (males 15%; females 13%), and sour taste (males/females 11%), while umami taste exposure was lowest (males/females 10%).

Strengths (+) and limitations (−) of all three methods for characterizing dietary taste patterns are given in Table 1 (panel B). The cluster method provided information about the prominent taste combinations in each diet and the extent to which each taste cluster contributed to total intake. However, since cluster formation depends on the data used, a direct comparison between diets is not possible. Taste amount and taste exposure, on the other hand, consider the same five basic tastes and fatty mouthfeel in all population groups, allowing direct comparison. Taste amount provided information about the intensity of each taste in the diet but does not consider in‐mouth exposure. Taste exposure allows consideration of both the intensity and duration of each taste experienced, providing a more comprehensive single measure of dietary taste or the taste of a diet. However, neither taste amount nor taste exposure allows representation of neutral (low‐taste‐intensity) foods. Considering these strengths and limitations, the cluster method was considered the most appropriate method to describe a country's dietary taste profile, while the taste exposure method was considered most appropriate for comparison purposes.

### 2.2 Primary Aim: To Characterize and Compare Dietary Taste Patterns Across Several Countries Dependent on Obesity Prevalence: France, the Netherlands, the UK, Australia, and the US

Having determined the most suitable methods for characterizing the dietary taste profile of a country in a manner that allows full description and cross‐country comparison, these methods were then applied to the five countries selected for varying obesity prevalence.

#### 2.2.1 Dietary Records

For the comparison across countries, national food consumption data from adults (18–65 years) were taken from: the third French Individual and National Food Consumption Survey (INCA3) 2014–2015 (Dubuisson et al. 2017), the Dutch National Food Consumption Survey (DNFCS) 2012–2016 (van Rossum et al. 2020), the combined seventh and eighth waves of the UK National Diet and Nutrition Survey (NDNS) 2014–2016 (Public Health England 2016), the NNPAS 2011–2012 of Australia (Australian Bureau of Statistics 2013), and the ninth US National Health and Nutrition Examination Survey (NHANES) 2015–2016 (National Center for Health Statistics 2018). For the INCA3, three nonconsecutive 24 h dietary recalls were conducted using the GloboDiet (IARC) software program. For the DNFCS, two nonconsecutive 24 h dietary recalls were conducted using the GloboDiet (IARC) software program. For the NDNS, participants completed a 4‐day food and drink diary. For the NHANES, two nonconsecutive 24 h dietary recalls were conducted using computerized software. Participant characteristics for each survey are provided in Table 3. Obesity prevalence for each sample differed slightly from the estimates cited in the Introduction, most plausibly due to the survey year, but the ordering of countries remains consistent.

**TABLE 3:** Participant characteristics (aged 18–65y) from the national consumption surveys.

	FR: INCA3	NL: DNFCS	UK: NDNS	AU: NNPAS	US: NHANES
Number (*N*)	1606	1508	1092	6415	4105
Sex (*N* (%))	M: 669 (42%); F: 937 (58%)	M: 744 (49%); F: 764 (51%)	M: 471 (43%); F: 621 (57%)	M: 3110 (48%); F: 3305 (52%)	M: 1973 (48%); F: 2132 (52%)
BMI, kg/m^2^ (mean ± SD)	25.4 ± 4.7	26.0 ± 5.2	27.3 ± 5.5	27.3 ± 5.6	29.6 ± 7.4
Percentage with obesity (%)	14.8	18.6	26.4	26.7	40.6
Age, years (range, or mean ± SD)	^a^ 18–44 (*n* = 780) 45–64 (*n* = 826)	39.3 ± 14.5	41.5 ± 13.8	42.0 ± 13.1	41.2 ± 14.1

Abbreviations: AU, Australia; BMI, body mass index; F, female; FR, France; M, male; NL, the Netherlands; SD, standard deviation; UK, the United Kingdom; US, the United States.

^a^ Age in INCA3 was recorded by group instead of in years.

#### 2.2.2 Taste Values and Dietary Characterization

Initially, the French food taste database (Martin et al. 2014) was applied to the French consumption data, the Dutch Taste, Fat and Texture Database (Mars et al. 2020) was applied to the consumption data of the Netherlands, and the Australian Sensory‐Diet Database was applied to the consumption data of the UK, Australia, and US, due to the similarities across these diets in foods consumed (Hendrie et al. 2022; Papanicolas et al. 2019). Untested foods were assigned taste and mouthfeel intensity values using “group mean”, as above. All foods were assigned to a texture category (see Table S4.1). Differences between countries in these analyses, however, could result from the database used; thus, to maintain greater consistency between countries, all analyses were also completed using taste intensity values from the Australian database. This latter analysis is presented here. Analyses based on initial database assignments are given in the Figures S4.1 and S4.2.

#### 2.2.3 Analysis

Diets were described using the cluster method and compared using “taste exposure”. To compare countries, separate basic linear model analyses were performed per taste and for fatty mouthfeel. Each model was adjusted for sex, BMI, grams consumed, and energy consumed to allow for these factors that may influence intake (Meiselman 2020). For each model, statistically defined outliers (1.5 × the interquartile range outside the first or third quartile) were removed, and Bonferroni‐corrected post hoc analyses were performed to investigate significant main effects. Analyses were performed using R statistical software (version 4.0.2) and relevant packages. A *p* < 0.05 was considered statistically significant.

## 3 Results

### 3.1 Dominant Taste Clusters

**FIGURE 1:**
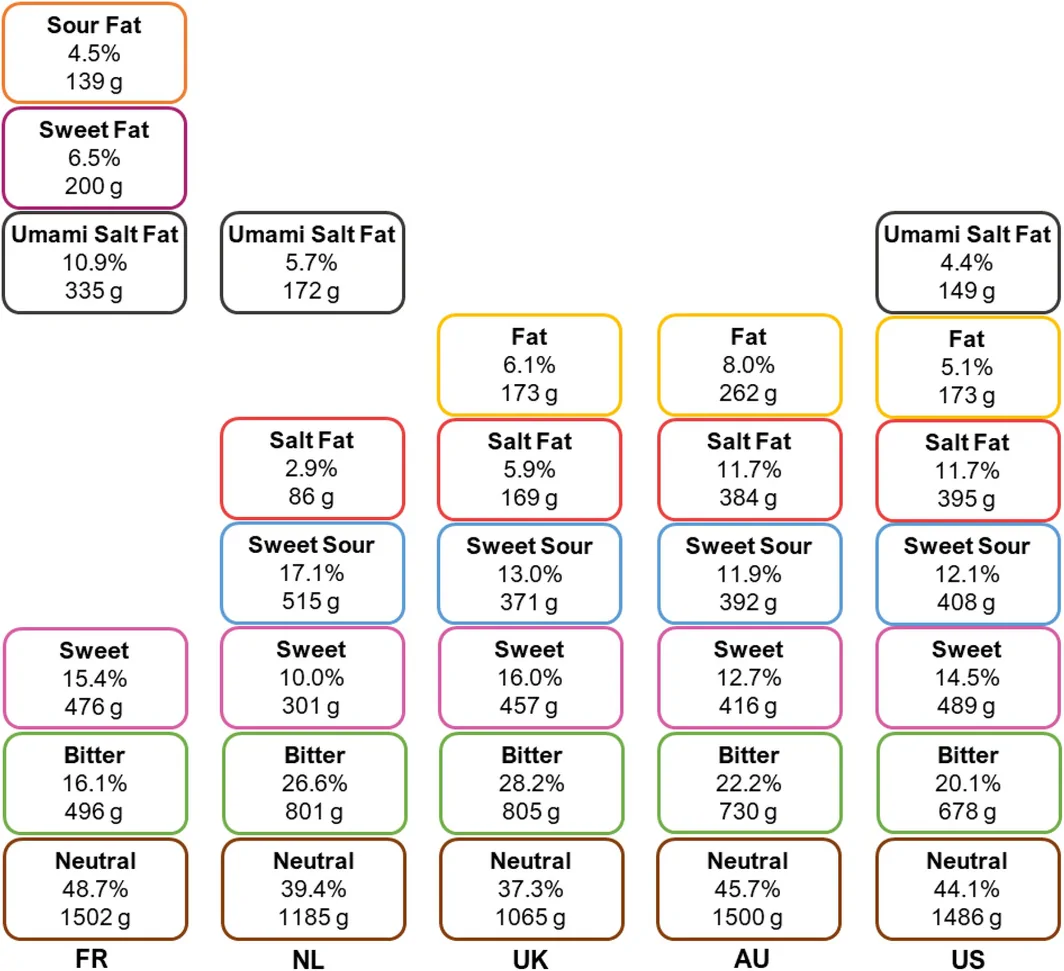
Taste clusters, their consumed quantities (in g) and percentage contributions (%) to the diets of France (FR), the Netherlands (NL), the United Kingdom (UK), Australia (AU) and the United States (US). Clusters are ordered based on relative contribution to dietary taste. Clusters are created per country, so direct comparison between countries is not possible.

Taste clusters for the diets of all five countries are given in Figure 1. Six clusters emerged for all countries, with the exception that the diet from the US resulted in seven clusters. All dietary profiles included a “Neutral”, “Bitter”, and “Sweet” cluster, with the “Neutral” cluster contributing the most to total dietary intake in all five countries. Further description of these profiles is provided in Tables S4.2 and S4.3. While all cluster analyses were undertaken on individual foods, Table S4.2 also includes the broader minor food (sub‐) groups in each cluster per country (with some duplication if individual foods from the same food (sub‐) group taste differently and so belong in different taste clusters). The differences between clusters for the different countries, in number, dominant taste, and contributing foods demonstrate the difficulty of comparing countries using this method.

### 3.2 Dietary Taste Exposure

**FIGURE 2:**
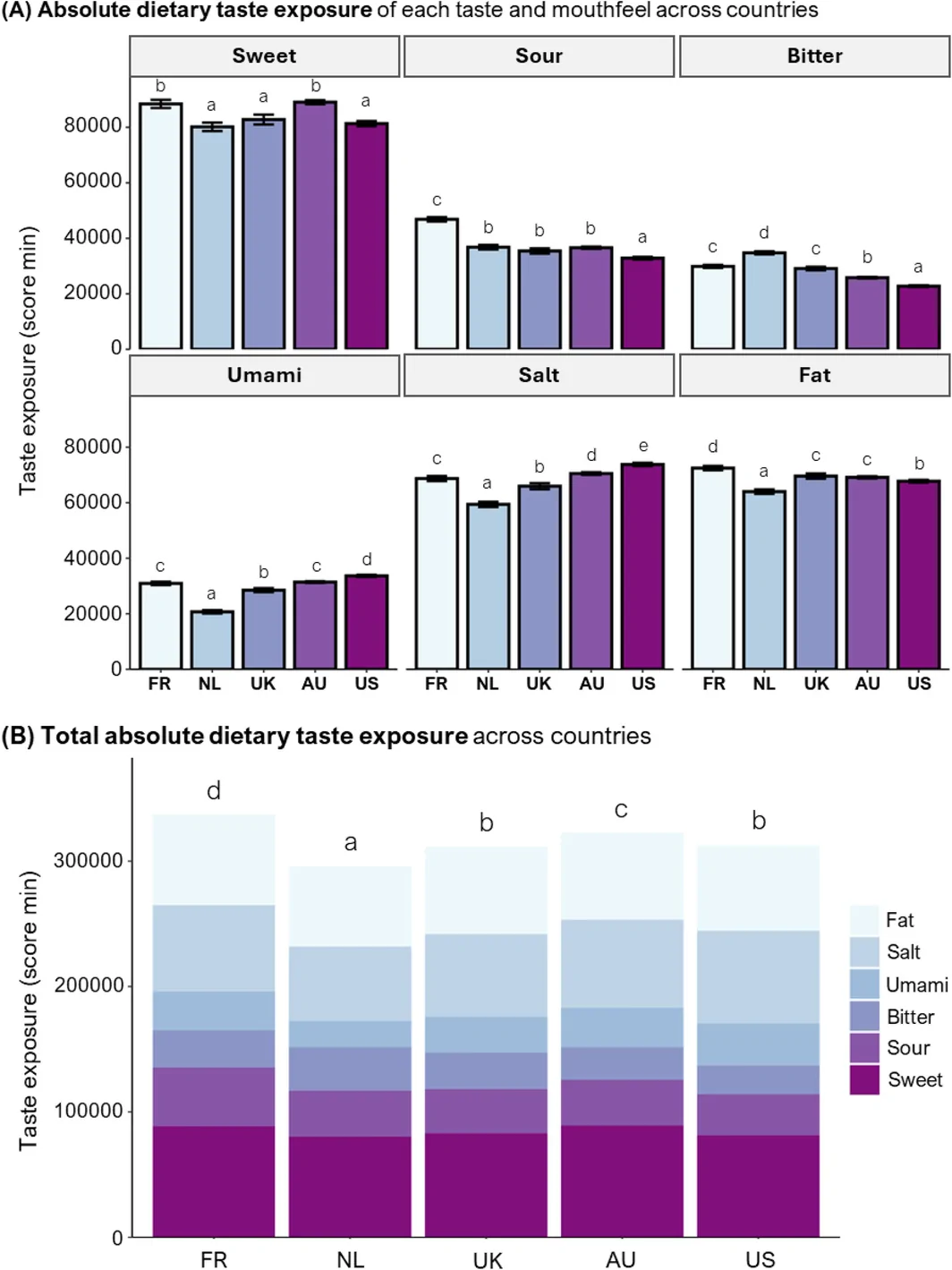
Absolute dietary taste exposure (A) of each basic taste (sweet, sour, bitter, umami, salt) and fatty mouthfeel (fat) and (B) summed for France (FR), the Netherlands (NL), the United Kingdom (UK), Australia (AU), and the United States (US). Within each taste or mouthfeel, using Bonferroni correction, bars with different letters are significantly different between countries at *p* ≤ 0.007, where “a” always represents the smallest value.

**FIGURE 3:**
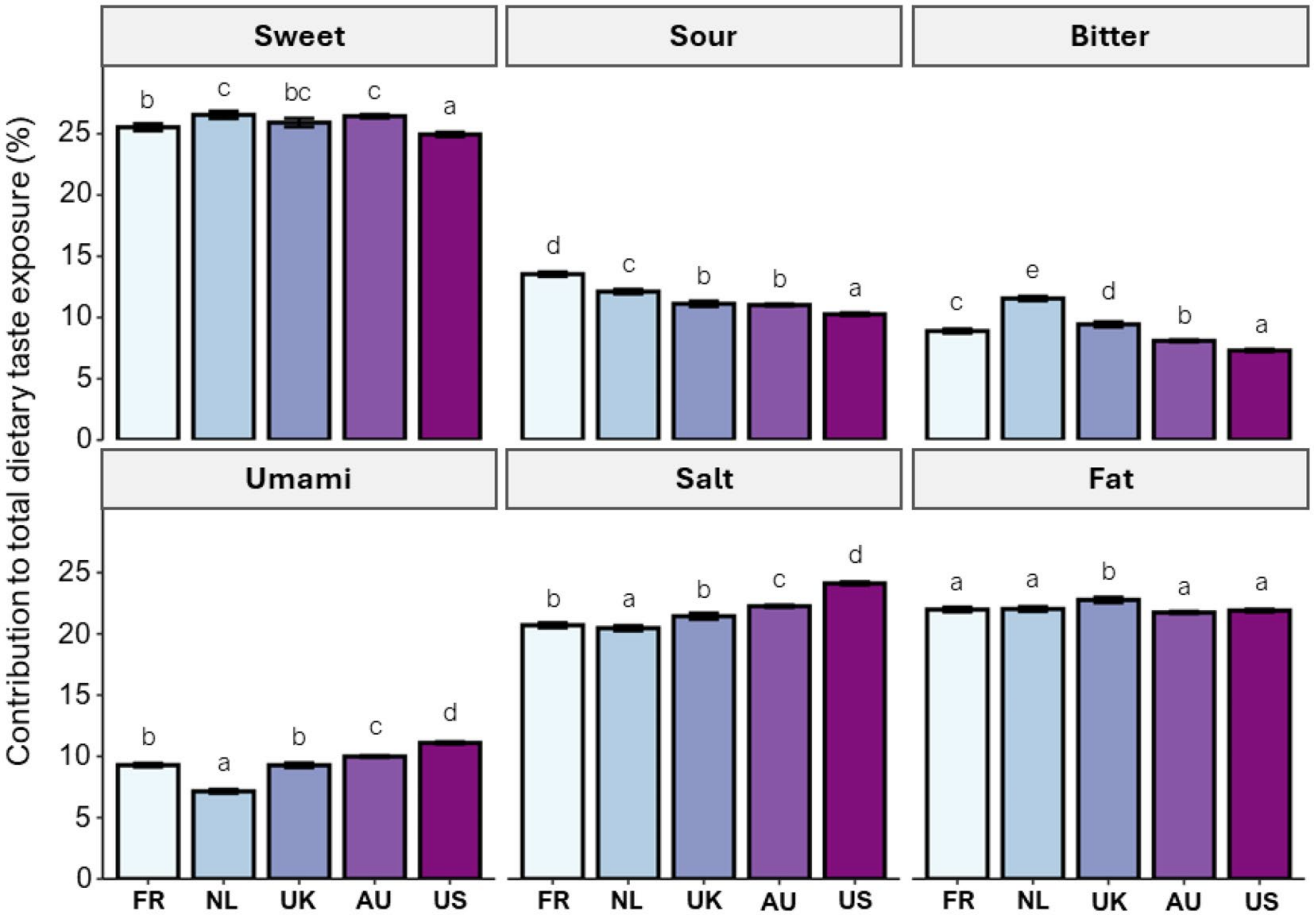
Percentage contribution of each basic taste (sweet, sour, bitter, umami, salt) and fatty mouthfeel (fat) to total exposure for France (FR), the Netherlands (NL), the United Kingdom (UK), Australia (AU), and the United States (US). Within each taste or mouthfeel, using Bonferroni correction, bars with different letters are significantly different between countries at *p* ≤ 0.0104, where “a” always represents the smallest value.

Exposure to each taste in the diets of each country (absolute values, total exposure, and proportion of total exposure) is presented in Figures 2 and 3. Values are given in Table S4.4. Total taste exposure was highest in the French diet and lowest in the Dutch diet (*R* ^2^ = 0.661, adjusted *R* ^2^ = 0.661, *F*(8,14,717) = 3593, *p* < 0.0001). For all countries, taste exposure was highest for sweet, salt, and fatty mouthfeel, both as absolute values and as a proportion of total taste exposure. Differences between countries were found in exposure to all tastes (smallest F(8,14,717) = 832, *p* < 0.001), but particularly in salt, bitter, umami, and fatty mouthfeel (smallest *F*(8,14,717) = 916, *p* < 0.001). For all five countries, sweet taste contributed the most to total exposure, with the lowest contribution in the US. Salt and fatty mouthfeel also contributed considerably, with the greatest contribution from salt in the US diet and from fatty mouthfeel in the UK diet. Bitter taste contributed least to total exposure; it was highest in the Netherlands. Umami and sour tastes also contributed minimally, with the highest contributions in the diets of the US and France, respectively.

## 4 Discussion

Considering known associations between taste and dietary intake, and between dietary patterns and obesity, this paper characterized and compared the dietary taste profiles of five countries with different rates of obesity: France, the Netherlands, the UK, Australia, and the US. To this aim, taste intensity data were combined with dietary intake data from national consumption surveys. The overall taste of the diet of each country was then described using a cluster approach to highlight dominant taste combinations, while taste exposure was used for direct comparisons between countries.

Several similarities were observed across the diets of all five countries. Neutral was the most prominent taste cluster in all countries, contributing 37.3%–48.7% of the weight consumed; a finding that likely reflects the low taste intensity of staple, high‐carbohydrate foods such as rice, cereals, pasta, and potatoes (Teo et al. 2022; van Langeveld et al. 2018; Cox, Hendrie, Lease, Rebuli, and Barnes 2018). Bitter was a distinct group in all countries, demonstrating coffee, tea and beer consumption (Cox, Hendrie, and Lease 2018). Sweet taste is represented in the form of sugar, reduced or low/no‐calorie sweeteners, and fruit, which can be consumed alone, in association with many neutral‐tasting foods, and in beverage form (Teo et al. 2022; van Langeveld et al. 2018; Cox, Hendrie, Lease, Rebuli, and Barnes 2018).

Similarities across countries are also seen where countries were directly compared using “taste exposure”. For all countries, taste exposure was highest for sweet taste, salt taste, and fatty mouthfeel, both as absolute values and as a proportion of total taste exposure, with sweet taste contributing the most, at around 25% of total taste exposure. While salt taste and fatty mouthfeel also contribute 20%–25% of total taste exposure, these three tastes were previously also reported to contribute most to the energy consumed from the Australian diet (Cox, Hendrie, Lease, Rebuli, and Barnes 2018). These findings testify to the known liking for all three tastes in humans (Appleton 2024; Beauchamp 2016; Drewnowski 1997; Leshem 2009), liking that has previously been linked to physiological benefit (Beauchamp 2016; Drewnowski 1997; Leshem 2009; Breslin 2013; Reed and Knaapila 2010). Sour, at around 10% of total taste exposure, and bitter and umami at 5%–10% of total taste exposure contribute less to all diets. Sour and bitter tastes are traditionally associated with spoilage, bacteria, and toxins, where consumption is not desirable (Breslin 2013; Reed and Knaapila 2010). Umami taste may also be associated with these food characteristics, for example, in blue cheeses, or is more often found in high‐protein foods, where again a high consumption may not be desirable (Breslin 2013; Reed and Knaapila 2010). Our findings thus may provide good evidence of the value of taste for safe consumption, and similarities across countries may be unsurprising.

Differences between countries, however, were also found. Total taste exposure was highest in the French diet and lowest in the Dutch diet. These findings may suggest differences in the consumption of neutral foods in these two countries, but the cluster analysis suggests that this is unlikely to be the case. These findings thus do suggest the higher consumption of foods with higher taste intensities in France.

Differences between countries were also noticeable in the contributions to total taste exposure from salt, umami, sour, and bitter taste. Total salt and umami taste exposure were highest in, and contributed most to, total taste exposure in the US diet. Conversely, salt taste and umami taste were low in the Dutch diet, while bitter taste was high. Sour taste was noticeably higher in the French diet. Considering these differences, it is interesting to note that the US also has the highest rates of obesity, while France and the Netherlands have the lowest (NCD Risk Factor Collaboration (NCD‐RisC) 2024). Thus, while no clear patterns with obesity prevalence are seen in total taste exposure, and sweet taste exposure is high in all countries, it is possibly the salt‐ and umami‐tasting dietary items that are contributing more to obesity. An analysis of the Dutch diet also finds associations between the consumption of salt, umami, and fat tastes and high body weight (van Langeveld et al. 2018). Specific food groups can be implicated—high‐protein and high‐fat foods, such as meat and related products, high‐salt, saturated‐fat foods, and fast‐food options, and the links between consumption of these food groups and obesity are well established (e.g., (Rosenheck 2008; Vergnaud et al. 2010)). Our findings add considerably to this existing work by suggesting that the taste of these products alongside their nutrient content may be important for excess energy intake.

The taste of a diet may have important implications for health risk via associations with consumption. It is noteworthy that the taste profiles of diets high and low in diet quality (Cox, Hendrie, and Lease 2018), and high and low in discretionary foods compared to core foods (Cox, Hendrie, Lease, Rebuli, and Barnes 2018), have previously been found to be similar (Cox, Hendrie, Lease, Rebuli, and Barnes 2018; Cox, Hendrie, and Lease 2018). Thus, there is some suggestion that dietary taste patterns can be maintained when moving to a healthier diet. Recommendations that consider dietary taste may facilitate adherence to such diets. The variation between countries found in our analyses suggests potential benefit to recommendations that are country‐specific.

Dietary taste patterns have previously been quantified in different ways (van Langeveld et al. 2018; Cox, Hendrie, Lease, Rebuli, and Barnes 2018), and the work presented here comprehensively considers these different approaches. The cluster method allows for a clear characterization of each diet but is specific to each dataset and does not allow comparisons between diets. Furthermore, using the cluster method, umami taste does not contribute significantly to the Australian or UK diet, while using taste exposure some contribution is found. The taste exposure method may be more sensitive while also allowing comparison between countries. The inclusion of oral processing rate in this measure also provides a novel extension to previous efforts to characterize dietary patterns and allows a more comprehensive assessment of dietary taste profiles. Use of weight consumed rather than energy or energy density also allows contributions from all items in the diet with tastants, including those with no energy, that is, low/no‐calorie‐sweetened beverages.

This work benefits from the preliminary investigations into methodology and the final use of both cluster and taste exposure methods to create national dietary taste profiles. Furthermore, to guard against differences based solely on the taste database used, the consumption data of all five countries were paired with the Australian Sensory‐Diet taste database. Limited differences were found in alternative analyses, but food products may differ and may taste different in different countries, hence, country‐specific databases may add nuance. In this respect it is unfortunate that the sensory profiles of several beverages are not included in the Australian Sensory‐Diet database, while different forms of these beverages may be consumed in different countries, for example, filtered black coffee, expresso, and café latte. These concerns highlight again the primary concern associated with using a group‐based approach for assigning taste values to all products within a food (sub)group. Our analyses are constrained by the sensory databases available. Fat taste and emerging “complex carbohydrate” and “kokumi” tastes (Keast et al. 2021) were not considered. Indeed, “starchy”, “cooked grain”, or “complex carbohydrate” taste (Keast et al. 2021) may more accurately describe the dominant tastes of the staple foods that we have described as “neutral” in taste. Similar limitations may also occur on an individual basis. Individual variation exists in taste perception (Appleton 2024), in rate of eating (Lasschuijt et al. 2021; Forde et al. 2017), in food preparation methods, and in consumption patterns where tastes interact (James et al. 2009; Trumbo et al. 2021), although none of these effects will differ systematically between countries. Differences based on demographic characteristics, such as sex and BMI, may also have affected our findings (van Langeveld et al. 2018; Cox, Hendrie, Lease, Rebuli, and Barnes 2018). Demographic differences between the national samples are small, but there are implications for our work if obesity is associated with reduced taste sensitivity or intensity perception (van Langeveld et al. 2018). In this situation, increasing body weight may be associated not only with a high consumption of salt, umami, and fat tastes, relative to other tastes, but also with greater consumption or greater consumption of the more intense‐tasting foods. We find no association between obesity prevalence at a country level and total taste exposure, but more nuanced analyses at an individual level would be of interest.

Our work will be of value through the elucidation of dietary taste patterns associated with health outcomes. A natural progression would be to utilize the above approaches to characterize and compare dietary taste patterns in different population groups, including those with health conditions. The analysis presented here is also notably cross‐sectional, while longitudinal associations with health outcomes would be informative.

## 5 Conclusion

To conclude, the taste profiles of the diets of France, the Netherlands, the UK, Australia, and the US were found to be largely similar, with the diets of all five countries comprising “neutral”, “bitter” and “sweet” taste clusters, and sweet taste exposure contributing the most to total taste exposure and umami contributing the least. Differences were found in total taste exposure and in the relative contributions of salt, umami, bitter, and sour tastes to national profiles, such that salt and umami tastes were highest in and contributed most to US diets, while bitter taste was highest in the Dutch diet and sour taste was highest in the French diet. Variation across the countries points to the importance of population‐specific research and recommendations.

## Author Contributions

**David N. Cox:** methodology, writing – review and editing. **Claudia S. Tang:** conceptualization, methodology, formal analysis, investigation, writing – original draft, writing – review and editing. **Monica Mars:** conceptualization, methodology, writing – review and editing, supervision, funding acquisition. **Gilly A. Hendrie:** investigation, writing – review and editing, methodology. **Katherine M. Appleton:** conceptualization, methodology, supervision, writing – review and editing, funding acquisition. **Kees de Graaf:** conceptualization, methodology, writing – review and editing, validation.

## Funding

This work was undertaken as part of a PhD studentship undertaken by Claudia S Tang, supervised by Monica Mars and Katherine M Appleton, funded by Bournemouth University, UK, and Wageningen University & Research, the Netherlands.

## Disclosure

Claudia S. Tang, Gilly A. Hendrie, David N. Cox have no competing interests to declare. Monica Mars, Kees de Graaf and Katherine M. Appleton have previously received research funding from a consortium of industry partners composed of the American Beverage Association, Arla Foods, Cargill R&D Center Europe BVBA, DSM‐Firmenich SA, International Sweeteners Association, SinoSweet Co. Ltd., Cosun Nutrition Center and Unilever Foods Innovation Center Wageningen. Monica Mars has also previously received research funding from Royal Cosun (sugar beet refinery) and Sensus (inulin producer) and has received expenses from ILSI Europe. Kees de Graaf is a member of the Global Nutrition Advisory Board of Mars Company. Kees de Graaf has received travel, hotel, and speaker remuneration from the International Sweeteners Association, and received speaker expenses from ILSI North America. Katherine M. Appleton has previously received research funding from ILSI North America, US, the Institute for the Advancement of Food and Nutrition Sciences (IAFNS), US, the International Sweeteners Association, BE, Ajinomoto Health and Nutrition North America Inc. US; and has current funding from The Coca Cola Company, US. Katherine M. Appleton has received speaker's expenses from EatWell Global, PepsiCo, US, and SEVA (The Hellenic Soft drinks Industry Association), Greece.

## Supporting information


**Table S1.1:** Average tastes and mouthfeel intensity values across all foods (*n* = 5648) based on (i) Best match, (ii) Group mean, and (iii) Group median.
**Table S1.2:** Mean differences in the taste intensity values between assignment methods across all untested foods.
**Figure S1.1:** Bland–Altman plots of the differences between best match and group mean, and differences between best match and group median; for all tastes and mouthfeel values.
**Table S2.1:** Statistics of various distance measures and clustering methods.
**Figure S2.1:** Scatterplots of clusters formed with different distance measures.
**Table S2.2:** Taste and mouthfeel values of six clusters formed by Ward's clustering method with.
(A) Euclidean, (B) Manhattan, (C) Maximum, and (D) Canberra distance measures.
**Table S2.3:** Minor food groups in each cluster formed by Ward's Linkage with either (A) Euclidean, (B) Manhattan, or (C) Maximum distance measures.
**Table S2.4:** Cluster differences between Euclidean, Manhattan, and Maximum distance measures.
**Table S2.5:** Taste and mouthfeel values alongside percentage contribution to total energy intake from each taste cluster using Ward's with Euclidean method, in (A) six, and (B) seven clusters.
**Table S3.1:** Standard for evaluating eating rate categories.
**Figure S3.1:** Sex differences in percentage contributions of taste clusters to amount eaten.
**Figure S3.2:** Sex differences in taste amounts from (A) whole diet, (B) food, and (C) beverage.
**Figure S3.3:** Sex differences in (A) taste exposures and (B) percentage contributions to total taste exposure.
**Table S4.1:** Percentage (and number) of foods in each texture category across the countries.
**Table S4.2:** Minor food groups from each country in each cluster.
**Table S4.3:** Energy contribution per taste across the countries calculated by summing the percentages of clusters in which the taste appears in.
**Table S4.4:** Estimated marginal means of absolute and percentage contributions of dietary taste exposure from nationally representative diets of France (FR), the Netherlands (NL), the United Kingdom (UK), the Australia (AU) and the United States (US).
**Figure S4.1:** Taste clusters, their average intake amounts (g) and percentage contribution (%) to the diets of France (FR) using the FR taste database, the Netherlands (NL) using the NL taste database, the United Kingdom (UK) using the Australian (AU) database, AU using the AU taste database, and the United States (US) using the AU database.
**Figure S4.2:** Stacked plots of each taste and mouthfeel in (A) absolute values and (B) percentage contribution to total dietary taste exposure for France (FR) using the FR taste database, the Netherlands (NL) using the NL taste database, the United Kingdom (UK) using the AU database, Australia (AU) using the AU taste database, and the United States (US) using the AU database. Bars labeled with different letters are different at p < 0.001, using Bonferroni‐corrected comparisons to compare total taste exposure, where ‘a’ always represents the smallest value.

## Data Availability

The data that support the findings of this study are available on request from the corresponding author. The data are not publicly available due to privacy or ethical restrictions.
