# Supplementary material for: Characterization and Comparison of Dietary Taste Patterns in Five Countries With Differing Obesity Prevalence
Source: Food Sci Nutr. 2026 Sep 16;14(9):e72307. doi: 10.1002/fsn3.72307 (PMC13583456; doi:10.1002/fsn3.72307)
Supplement: Supplementary file 1 [file FSN3-14-e72307-s001.docx]

**Characterisation and comparison of dietary taste patterns in five countries with differing obesity prevalence**

Claudia S. Tang, Gilly A. Hendrie, David N. Cox, Monica Mars, Kees de Graaf and Katherine M. Appleton

**Supplementary Materials**

1. **Assignment methods – ‘Best match’, group mean and group median**

- Table S1.1. Average tastes and mouthfeel intensity values across all foods (n=5648) based on i) Best match, ii) Group mean and iii) Group median.
- Table S1.2. Mean differences in the taste intensity values between assignment methods across all untested foods.
- Figure S1.1. Bland-Altman plots of the differences between best match and group mean, and differences between best match and group median; for all tastes and mouthfeel values

2. **Characterisation methods – Cluster**

- Table S2.1. Statistics of various distance measures and clustering methods
- Figure S2.1. Scatterplots of clusters formed with different distance measures
- Table S2.2. Taste and mouthfeel values of six clusters formed by Ward’s clustering method with
  (A) Euclidean, (B) Manhattan, (C) Maximum and (D) Canberra distance measures
- Table S2.3. Minor food groups in each cluster formed by Ward’s Linkage with either A) Euclidean, B) Manhattan or C) Maximum distance measures
- Table S2.4. Cluster differences between Euclidean, Manhattan and Maximum distance measures
- Table S2.5. Taste and mouthfeel values alongside percentage contribution to total energy intake from each taste cluster using Ward’s with Euclidean method, in (A) six, and (B) seven clusters

3. **Characterisation methods – Taste amount and taste exposure**

- Table S3.1. Standard for evaluating eating rate categories
- Figure S3.1. Sex differences in percentage contributions of taste clusters to amount eaten
- Figure S3.2. Sex differences in taste amounts from (A) whole diet, (B) food, and (C) beverage
- Figure S3.3. Sex differences in (A) taste exposures and (B) percentage contributions to total taste exposure

4. **Cross-country comparisons**

- Table S4.1. Percentage (and number) of foods in each texture category across the countries
- Table S4.2. Minor food groups from each country in each cluster
- Table S4.3. Energy contribution per taste across the countries calculated by summing up percentages of clusters in which the taste appears in
- Table S4.4. Estimated marginal means of absolute and percentage contributions of dietary taste exposure from nationally representative diets of France (FR), the Netherlands (NL), the United Kingdom (UK), Australia (AU) and the United States (US)
- Figure S4.1. Taste clusters, their average intake amounts (g) and percentage contribution (%) to the diets of France (FR) using the FR taste database, the Netherlands (NL) using the NL taste database, the United Kingdom (UK) using the Australian (AU) database, AU using the AU taste database, and the United States (US) using the AU database.
- Figure S4.2. Stacked plots of each taste and mouthfeel in (A) absolute values and (B) percentage contribution to total dietary taste exposure for France (FR) using the FR taste database, the Netherlands (NL) using the NL taste database, the United Kingdom (UK) using the AU database, Australia (AU) using the AU taste database and the United States (US) using the AU database. Bars labelled with different letters are different at *p* < .001, using Bonferroni-corrected comparisons to compare total taste exposure, where ‘a’ always represents the smallest value.

Supplementary Table S1.1. Average tastes and mouthfeel intensity values across all foods (n=5648) based on i) Best match, ii) Group^1^ mean and iii) Group^1^ median.

| Intensity value of/based on | **Best match** | **Group mean** | **Group median** |
| --- | --- | --- | --- |
| Sweet taste | 23.1± 21.1 | 22.5 ± 18.3 | 22.2 ± 19.8 |
| Sour taste | 7.2 ± 10.4 | 8.2 ± 9.7 | 7.3 ± 9.7 |
| Bitter taste | 4.1 ± 8.4 | 4.5 ± 7.4 | 3.6 ± 8.1 |
| Umami taste | 8.5 ± 10.5 | 8.2 ± 7.8 | 7.3 ± 7.8 |
| Salt taste | 17.7 ± 15.0 | 17.8 ± 13.1 | 16.6 ± 13.8 |
| Fatty mouthfeel | 21.2 ± 15.0 | 20.2 ± 13.0 | 19.0 ± 13.2 |

^1^ Group refers to AUSNUT (sub-)group where each taste and mouthfeel were averaged and assigned to all untested foods within that (sub-)group.

Supplementary Table S1.2. Mean differences in the taste intensity values between assignment methods across all untested foods.

|  |  | **Mean paired difference ± SD** | **95% CI** | ***t*(5647)** ^1^ | ***p*** |
| --- | --- | --- | --- | --- | --- |
| **Best match vs.**  **group mean** | Sweet taste | 0.42 ± 8.75 | [0.19, 0.65] | 3.58 | < .001 |
|  | Sour taste | -1.19 ± 6.59 | [-1.36, -1.02] | -13.5 | < .001 |
|  | Bitter taste | -0.58 ± 5.47 | [-0.72, -0.43] | -7.92 | < .001 |
|  | Umami taste | 0.31 ± 6.71 | [0.13, 0.48] | 3.43 | < .001 |
|  | Salt taste | -0.15 ± 8.63 | [-0.38, 0.07] | -1.32 | .188 |
|  | Fatty mouthfeel | 0.97 ± 8.02 | [0.76, 1.18] | 9.09 | < .001 |
| **Best match vs.**  **group median** | Sweet taste | 0.97 ± 8.52 | [0.74, 1.19] | 8.35 | < .001 |
|  | Sour taste | -0.11 ± 6.10 | [-0.27, 0.05] | -1.34 | .180 |
|  | Bitter taste | 0.54 ± 4.31 | [0.42, 0.65] | 9.16 | < .001 |
|  | Umami taste | 1.17 ± 7.42 | [0.97, 1.34] | 11.6 | < .001 |
|  | Salt taste | 1.12 ± 9.04 | [0.88, 1.36] | 9.16 | < .001 |
|  | Fatty mouthfeel | 2.17 ± 8.26 | [1.95, 2.39] | 19.4 | < .001 |

SD: standard deviation. CI: confidence interval. ^1^ Total number of foods.

Supplementary Figure S1.1. Bland-Altman plots (Giavarina, D. (2015). Understanding Bland Altman analysis. *Biochemia Medica*, *25*(2), 141–151. https://doi.org/10.11613/bm.2015.015) of the differences between best match and group mean, and differences between best match and group median; for all tastes and mouthfeel values.

Supplementary Table S2.1. Statistics of various distance measures and clustering methods.

|  |  | **Clustering method** | | | |
| --- | --- | --- | --- | --- | --- |
|  |  | 1. Ward’s | 2. Average | 3. Weighted | 4. Centroid |
| **Distance measure** | i) Euclidean | c = 0.519 | c = 0.839 | c = 0.693 | c = 0.774 |
|  |  | λ = 0.00258 | - | - | - |
|  | ii) Manhattan | c = 0.563 | c = 0.750 | c = 0.616 | c = 0.732 |
|  |  | λ = 0.00372 | - | λ = 0.0386 | - |
|  | iii) Maximum | c = 0.536 | c = 0.871 | c = 0.625 | c = 0.831 |
|  |  | λ = 0.00381 | - | - | - |
|  | iv) Canberra | c = 0.677 | c = 0.750 | c = 0.714 | c = 0.572 |
|  |  | λ = 0.0196 | λ = 0.196 | - | - |

c: Cophenetic Correlation Coefficient. λ: Wilk’s Lambda. Note: The standardised data produced similar results for Euclidean, Minkowski and Pearson distance measures. Since Minkowski is a generalisation of the Euclidean and Manhattan distance measures (Witten et al., 2016), its results were excluded.

These values inform how well the pairwise distances of resultant clusters match with those between the original unmodeled datapoints (Akogul, S., & Erisoglu, M. (2017). An Approach for Determining the Number of Clusters in a Model-Based Cluster Analysis. *Entropy*, *19*(9), 452. https://doi.org/10.3390/e19090452; Saraçli, S., Doǧan, N., & Doǧan, I. (2013). Comparison of hierarchical cluster analysis methods by cophenetic correlation. *Journal of Inequalities and Applications*, *2013*(1), 1–8. https://doi.org/10.1186/1029-242x-2013-203), and were calculated based on six clusters specification, since there are five tastes and one mouthfeel variable.

Supplementary Figure S2.1. Scatterplot of six clusters formed by Ward’s clustering method with (i) Euclidean, (ii) Manhattan, (iii) Maximum, and (iv) Canberra distance measures.

Among clustering methods, the λ values of Average and Weighted clustering were considerably higher than values from Ward’s clustering, hence, Ward’s clustering method was chosen. Among distance measures, Canberra distance measure resulted in two similar clusters and remaining clusters did not have as high values as compared to the other distance measures; hence, it was excluded. Euclidean, Manhattan and Maximum distance measures produced similar dominant tastes in each cluster, and Euclidean produced the most logical clusters, thus, it was chosen.

Supplementary Table S2.2. Taste and mouthfeel values of six clusters formed by Ward’s clustering method with (A) Euclidean, (B) Manhattan, (C) Maximum and (D) Canberra distance measures.

| **A) Euclidean** (mean ± SD) | | | | | | |
| --- | --- | --- | --- | --- | --- | --- |
| No. of foods | **n=78** | **n=834** | **n=1007** | **n=357** | **n=1456** | **n=814** |
| Sweet taste | 10.6±5.5 | 13.6±7.6 | 52.6±10.1 | 32.4±12.5 | 15.8±5.9 | 8.7±6.5 |
| Sour taste | 5.0±3.7 | 5.9±4.8 | 7.1±5.9 | 36.4±10.0 | 7.7±5.6 | 2.8±1.7 |
| Bitter taste | 55.4±4.4^1^ | 7.8±6.7 | 3.3±2.6 | 6.4±6.5 | 2.5±2.2 | 2.4±3.2 |
| Umami taste | 0.7±0.3 | 3.1±1.9 | 0.7±0.4 | 2.2±2.7 | 17.0±6.0 | 8.0±5.6 |
| Salt taste | 1.6±0.8 | 10.4±7.0 | 9.0±4.4 | 9.3±10.3 | 34.6±9.0 | 13.6±6.4 |
| Fatty mouthfeel | 6.6±4.4 | 7.4±4.8 | 17.0±10.3 | 12.0±15.4 | 26.3±9.8 | 29.1±13.6 |
| **B) Manhattan** (mean ± SD) | | | | | | |
| No. of foods | **n=78** | **n=813** | **n=1070** | **n=319** | **n=1152** | **n=1108** |
| Sweet taste | 10.6±5.5 | 13.5±7.5 | 51.2±11.4 | 33.1±12.4 | 17.6±5.3 | 8.1±4.1 |
| Sour taste | 5.02±3.7 | 5.9±4.7 | 7.1±5.8 | 35.2±8.7 | 10.4±9.0 | 2.6±1.6 |
| Bitter taste | 55.4±4.4 | 8.1±6.8 | 3.2±2.6 | 6.7±6.9 | 2.6±2.2 | 2.3±2.8 |
| Umami taste | 0.7±0.3 | 3.1±2.0 | 0.7±0.6 | 1.6±1.8 | 18.4±5.8 | 9.3±5.1 |
| Salt taste | 1.6±0.8 | 10.4±7.0 | 8.9±4.4 | 6.4±5.5 | 35.3±8.6 | 19.8±11.2 |
| Fatty mouthfeel | 6.6±4.4 | 7.4±4.9 | 17.9±10.6 | 10.4±14.9 | 28.4±9.4 | 25.5±13.4 |
| **C) Maximum** (mean ± SD) | | | | | | |
| No. of foods | **n=78** | **n=1438** | **n=1009** | **n=466** | **n=1474** | **n=81** |
| Sweet taste | 10.6±5.5 | 10.6±7.4 | 52.1±11.1 | 30.4±12.6 | 15.7±5.8 | 8.7±2.5 |
| Sour taste | 5.0±3.7 | 3.8±3.1 | 6.4±5.4 | 31.0±12.7 | 7.9±6.3 | 4.7±2.4 |
| Bitter taste | 55.4±4.4 | 4.5±0.4 | 3.4±2.3 | 8.6±8.8 | 2.6±2.6 | 2.3±4.9 |
| Umami taste | 0.7±0.3 | 6.0±5.0 | 0.8±0.6 | 1.9±2.4 | 16.9±3.1 | 2.4±1.6 |
| Salt taste | 1.6±0.8 | 11.7±5.4 | 9.1±4.2 | 7.3±8.5 | 34.6±9.0 | 26.2±10.1 |
| Fatty mouthfeel | 6.6±4.4 | 16.6±9.8 | 17.0±10.1 | 9.8±14.1 | 26.3±10.1 | 66.1±4.6 |
| **D) Canberra** (mean ± SD) | | | | | | |
| No. of foods | **n=1128** | **n=965** | **n=410** | **n=258** | **n=1366** | **n=419** |
| Sweet taste | 10.8±6.2 | 48.6±12.5 | 46.0±12.8 | 18.0±5.8 | 15.9±5.9 | 5.5±1.0 |
| Sour taste | 5.9±9.7 | 6.6±0.8 | 24.5±10.6 | 17.0±9.4 | 7.6±7.3 | 2.5±0.4 |
| Bitter taste | 10.0±14.4 | 3.1±3.3 | 5.2±3.8 | 7.0±2.5 | 2.2±1.5 | 2.1±0.5 |
| Umami taste | 2.7±2.3 | 8.8±8.3 | 1.2±1.2 | 9.4±8.0 | 17.0±5.7 | 12.9±2.8 |
| Salt taste | 11.3±9.7 | 9.6±3.9 | 5.0±3.7 | 24.4±10.9 | 34.4±8.8 | 12.8±2.5 |
| Fatty mouthfeel | 15.0±17.3 | 21.1±9.1 | 3.0±3.5 | 14.3±6.8 | 27.1±9.8 | 25.8±1.4 |

^1^ Underlined values that are highest within that cluster. SD: standard deviation.

Euclidean, Manhattan and Maximum distance measures produced similar dominant tastes in each cluster. In contrast, Canberra distance measure resulted in two similar clusters and remaining clusters did not have as high values as compared to the other distance measures; hence, it was excluded.

Supplementary Table S2.3. Minor food groups in each cluster formed by Ward’s Linkage with either A) Euclidean, B) Manhattan or C) Maximum distance measures.

|  | **A) Euclidean** | **B) Manhattan** | **C) Maximum** |
| --- | --- | --- | --- |
| **Cluster 1:**  **Bitter** | Tea, Coffee and coffee substitutes, Beers | Tea, Coffee and coffee substitutes, Beers | Tea, Coffee and coffee substitutes, Beers |
| **Cluster 2:**  **Neutral** | Tea, Waters, municipal and bottled, unflavoured, Flours and other cereal grains and starches, Regular breads, and bread rolls, English-style muffins, flat breads, and savoury and sweet breads, Pasta and pasta products (without sauce), Breakfast cereals, ready to eat, Savoury biscuits, Dry soup mix, Seeds and seed products, Nuts and nut products, Gravies and savoury sauces, Cabbage, cauliflower and similar brassica vegetables, Carrot and similar root vegetables, Leaf and stalk vegetables, Peas and beans, Tomato and tomato products, Other fruiting vegetables, Other vegetables and vegetable combinations, Dishes where vegetable is the major component, Mature legumes and pulses, Mature legume and pulse products and dishes, Spirits, Other alcoholic beverages, Intense sweetening agents, Herbs, spices, seasonings and stock cubes | Tea, Waters, municipal and bottled, unflavoured, Flours and other cereal grains and starches, Regular breads, and bread rolls, English-style muffins, flat breads, and savoury and sweet breads, Pasta and pasta products (without sauce), Breakfast cereals, ready to eat, Savoury biscuits, Dry soup mix, Nuts and nut products, Gravies and savoury sauces, Cabbage, cauliflower and similar brassica vegetables, Carrot and similar root vegetables, Leaf and stalk vegetables, Peas and beans, Tomato and tomato products, Other fruiting vegetables, Other vegetables and vegetable combinations, Dishes where vegetable is the major component, Mature legumes and pulses, Mature legume and pulse products and dishes, Spirits, Other alcoholic beverages, Intense sweetening agents, Herbs, spices, seasonings and stock cubes | Waters, municipal and bottled, unflavoured, Flours and other cereal grains and starches, Regular breads, and bread rolls, English-style muffins, flat breads, and savoury and sweet breads, Pasta and pasta products (without sauce), Breakfast cereals, ready to eat, Breakfast cereals, hot porridge style, Savoury biscuits, Pastries, Mixed dishes where cereal is the major ingredient, Batter-based products, Eggs, Dishes where egg is the major ingredient, Beef, sheep and pork, unprocessed, Poultry and feathered game, Dairy milk (cow, sheep and goat), Cream, Cheese, Dairy milk substitutes, unflavoured, Seeds and seed products, Nuts and nut products, Potatoes, Cabbage, cauliflower and similar brassica vegetables, Carrot and similar root vegetables, Leaf and stalk vegetables, Peas and beans, Other fruiting vegetables, Other vegetables and vegetable combinations.Dishes where vegetable is the major component, Mature legumes and pulses, Mature legume and pulse products and dishes, Intense sweetening agents, Herbs, spices, seasonings and stock cubes |
| **Cluster 3:**  **Sweet** | Fruit and vegetable juices, and drinks, Soft drinks, and flavoured mineral waters, Other beverage flavourings and prepared beverages, English-style muffins, flat breads, and savoury and sweet breads, Breakfast cereals, ready to eat, Sweet biscuits, Cakes, muffins, scones, cake-type desserts, Pastries, Batter-based products, Pome fruit, Stone fruit, Tropical and subtropical fruit, Other fruit, Mixtures of two or more groups of fruit, Dried fruit, preserved fruit, Mixed dishes where fruit is the major component, Frozen milk products, Custards, Other dishes where milk or a milk product is the major component, Flavoured milks and milkshakes, Dairy milk substitutes, flavoured, Soy-based ice confection, Carrot and similar root vegetables, Other fruiting vegetables, Sugar, honey and syrups, Jam and lemon spreads, chocolate spreads, sauces, Dishes and products other than confectionery where sugar is the major component, Chocolate and chocolate-based confectionery, Fruit, nut and seed-bars, Muesli or cereal style bars, Other confectionery, Formula dietary foods | Fruit and vegetable juices, and drinks, Soft drinks, and flavoured mineral waters, Electrolyte, energy and fortified drinks, Other beverage flavourings and prepared beverages, English-style muffins, flat breads, and savoury and sweet breads, Breakfast cereals, ready to eat, Sweet biscuits, Cakes, muffins, scones, cake-type desserts, Pastries, Batter-based products, Pome fruit, Stone fruit, Tropical and subtropical fruit, Other fruit, Mixtures of two or more groups of fruit, Dried fruit, preserved fruit, Mixed dishes where fruit is the major component, Dairy milk (cow, sheep and goat), Frozen milk products, Custards, Other dishes where milk or a milk product is the major component, Flavoured milks and milkshakes, Dairy milk substitutes, unflavoured, Dairy milk substitutes, flavoured, Soy-based ice confection, Soy-based yoghurts, Carrot and similar root vegetables, Other fruiting vegetables, Sugar, honey and syrups, Jam and lemon spreads, chocolate spreads, sauces, Dishes and products other than confectionery where sugar is the major component, Chocolate and chocolate-based confectionery, Fruit, nut and seed-bars, Muesli or cereal style bars, Other confectionery, Formula dietary foods | Soft drinks, and flavoured mineral waters, Other beverage flavourings and prepared beverages, English-style muffins, flat breads, and savoury and sweet breads, Breakfast cereals, ready to eat, Sweet biscuits, Cakes, muffins, scones, cake-type desserts, Pastries, Batter-based products, Tropical and subtropical fruit, Mixtures of two or more groups of fruit, Dried fruit, preserved fruit, 169Frozen milk products, Custards, Other dishes where milk or a milk product is the major component, Flavoured milks and milkshakes, Dairy milk substitutes, flavoured, Soy-based ice confection, Carrot and similar root vegetables, Other fruiting vegetables, Sugar, honey and syrups, Jam and lemon spreads, chocolate spreads, sauces, Dishes and products other than confectionery where sugar is the major component, Chocolate and chocolate-based confectionery, Fruit, nut and seed-bars, Muesli or cereal style bars, Other confectionery, Formula dietary foods |
| **Cluster 4:**  **Sweet & Sour** | Fruit and vegetable juices, and drinks, Cordials, Soft drinks, and flavoured mineral waters, Electrolyte, energy and fortified drinks, Pome fruit, Berry fruit, Citrus fruit, Stone fruit, Other fruit, Dried fruit, preserved fruit, Yoghurt, Cream, Cheese, Soy-based yoghurts, Pickles, chutneys and relishes, Salad dressings, Wines, Cider and perry | Fruit and vegetable juices, and drinks, Cordials, Electrolyte, energy and fortified drinks, Pome fruit, Berry fruit, Citrus fruit, Stone fruit, Other fruit, Dried fruit, preserved fruit, Yoghurt, Cream, Cheese, Salad dressings, Tomato and tomato products, Wines, Spirits, Cider and perry | Tea, Fruit and vegetable juices, and drinks, Cordials, Electrolyte, energy and fortified drinks, Pome fruit, Berry fruit, Citrus fruit, Stone fruit, Tropical and subtropical fruit, Other fruit, Dried fruit, preserved fruit, Yoghurt, Cream, Cheese, Soy-based yoghurts, Gravies and savoury sauces, Salad dressings, Leaf and stalk vegetables, Tomato and tomato products, Other fruiting vegetables, Wines, Spirits, Cider and perry, Other alcoholic beverages |
| **Cluster 5:**  **Salt & Fat** | Regular breads, and bread rolls, Pasta and pasta products (without sauce), Pasta and pasta products (without sauce), Savoury biscuits, Pastries, Mixed dishes where cereal is the major ingredient, Fin fish, Crustacea and molluscs, Other sea and freshwater foods, Packed (commercially sterile) fish and seafood, Fish and seafood products, Mixed dishes with fish or seafood as the major component, Beef, sheep and pork, unprocessed, Mammalian game meats, Organ meats and offal, products and dishes, Sausages, frankfurts and saveloys, Processed meat, Mixed dishes where beef, sheep, pork or mammalian game is the major component, Mixed dishes where sausage, bacon, ham or other processed meat is the major component, Mixed dishes where poultry or feathered game is the major component, Cheese, Meat substitutes, Dishes where meat substitutes are the major component, Soup, homemade from basic ingredients, Soup, prepared from dry soup mix, Canned condensed soup (unprepared), Soup, commercially sterile, prepared from condensed or sold ready to eat, Soup, not commercially sterile, purchased ready to eat, Nuts and nut products, Gravies and savoury sauces, Dips, Potatoes, Tomato and tomato products, Other fruiting vegetables, Dishes where vegetable is the major component, Potato snacks, Corn snacks, Extruded or reformed snacks, Other snacks, Yeast, and yeast vegetable or meat extracts | English-style muffins, flat breads, and savoury and sweet breads, Pasta and pasta products (without sauce), Savoury biscuits, Pastries, Mixed dishes where cereal is the major ingredient, Mixed dishes with fish or seafood as the major component, Sausages, frankfurts and saveloys, Processed meat, Mixed dishes where beef, sheep, pork or mammalian game is the major component, Mixed dishes where sausage, bacon, ham or other processed meat is the major component, Mixed dishes where poultry or feathered game is the major component, Cheese, Meat substitutes, Dishes where meat substitutes are the major component, Soup, homemade from basic ingredients, Soup, prepared from dry soup mix, Canned condensed soup (unprepared), Soup, commercially sterile, prepared from condensed or sold ready to eat, Soup, not commercially sterile, purchased ready to eat, Gravies and savoury sauces, Pickles, chutneys and relishes, Salad dressings, Dips, Tomato and tomato products, Dishes where vegetable is the major component, Potato snacks, Extruded or reformed snacks, Other snacks, Yeast, and yeast vegetable or meat extracts | Regular breads, and bread rolls, Pasta and pasta products (without sauce), Savoury biscuits, Pastries, Mixed dishes where cereal is the major ingredient, Fin fish, Crustacea and molluscs, Other sea and freshwater foods, Packed (commercially sterile) fish and seafood, Fish and seafood products, Mixed dishes with fish or seafood as the major component, Mammalian game meats, Organ meats and offal, products and dishes, Sausages, frankfurts and saveloys, Processed meat, Mixed dishes where beef, sheep, pork or mammalian game is the major component, Mixed dishes where sausage, bacon, ham or other processed meat is the major component, Mixed dishes where poultry or feathered game is the major component, Cheese, Meat substitutes, Dishes where meat substitutes are the major component, Soup, homemade from basic ingredients, Soup, prepared from dry soup mix, Canned condensed soup (unprepared), Soup, commercially sterile, prepared from condensed or sold ready to eat, Soup, not commercially sterile, purchased ready to eat, Nuts and nut products, Gravies and savoury sauces, Pickles, chutneys and relishes, Dips, Potatoes, Other fruiting vegetables, Dishes where vegetable is the major component, Potato snacks, Corn snacks, Extruded or reformed snacks, Other snacks, Herbs, spices, seasonings and stock cubes |
| **Cluster 6:**  **Fat** | Breakfast cereals, hot porridge style, Pastries, Batter-based products, Butters, Dairy blends, Margarine and table spreads, Plant oils, Unspecified fats, Fish and seafood products, Eggs, Dishes where egg is the major ingredient, Beef, sheep and pork, unprocessed, Poultry and feathered game, Dairy milk (cow, sheep and goat), Cream, Dairy milk substitutes, unflavoured, Soup, homemade from basic ingredients, Nuts and nut products, Potatoes, Other fruiting vegetables | Flours and other cereal grains and starches, Regular breads, and bread rolls, Pasta and pasta products (without sauce), Breakfast cereals, ready to eat, Breakfast cereals, hot porridge style, Savoury biscuits, Pastries, Mixed dishes where cereal is the major ingredient, Batter-based products, Butters, Dairy blends, Margarine and table spreads, Plant oils, Fin fish, Crustacea and molluscs, Other sea and freshwater foods, Packed (commercially sterile) fish and seafood, Fish and seafood products, Eggs, Dishes where egg is the major ingredient, Beef, sheep and pork, unprocessed, Mammalian game meats, Poultry and feathered game, Organ meats and offal, products and dishes, Processed meat, Mixed dishes where poultry or feathered game is the major component, Dairy milk (cow, sheep and goat), Cream, Soup, homemade from basic ingredients, Nuts and nut products, Potatoes, Other fruiting vegetables, Mature legume and pulse products and dishes, Corn snacks, Other snacks, Yeast, and yeast vegetable or meat extracts | Butters, Dairy blends, Margarine and table spreads, Plant oils, Unspecified fats, Nuts and nut products |

Across Euclidean, Manhattan and Maximum distance measures, Cluster 1 (n=78) consistently had the highest value in bitter taste and was named ‘Bitter’. Cluster 2 (n=813-1438) consistently had low values in all tastes and mouthfeel and was named ‘Neutral’. Cluster 3 (n=1007-1070) consistently had the highest value in sweet taste and was named ‘Sweet’. Cluster 4 (n=319-466) consistently had the highest values in both sweet and sour tastes, hence it was named ‘Sweet and Sour’. Cluster 5 (n=1152-1474) consistently had the highest values in salt taste and fatty mouthfeel, hence it was named ‘Salt and Fat’. Lastly, Cluster 6 (n=81-1108) consistently had the highest value in fatty mouthfeel and was named ‘Fat’.

Supplementary Table S2.4. Cluster differences: Euclidean, Manhattan and Maximum distance measures.

| **Food groups** | **Euclidean** | **Manhattan** | **Maximum** |
| --- | --- | --- | --- |
| Tea | Bitter, Neutral | Bitter, Neutral | Bitter, Sweet & Sour |
| Pastries | Sweet, Salt & Fat, Fat | Sweet, Salt & Fat, Fat | Neutral, Sweet |
| Batter-based products | Sweet, Fat | Sweet, Fat | Neutral, Sweet |
| Savoury biscuits | Neutral, Salt & Fat | Neutral, Salt & Fat, Fat | Neutral, Salt & Fat |
| Corn snacks | Salt & Fat | Fat | Salt & Fat |
| Other snacks | Salt & Fat | Fat | Salt & Fat |
| Regular breads & bread rolls | Neutral, Salt & Fat | Neutral, Fat | Neutral, Salt & Fat |
| Flour, other cereal grain, starch | Neutral | Neutral, Fat | Neutral |
| Cream | Sweet & Sour, Fat | Sweet & Sour, Fat | Neutral, Sweet & Sour |
| Dairy milk (cow, sheep & goat) | Sweet, Fat | Sweet, Fat | Neutral, Sweet |
| Mixed dishes where cereal is the major ingredient | Salt & Fat | Salt & Fat, Fat | Neutral |
| Eggs | Fat | Fat | Neutral |
| Dishes where egg is the major ingredient | Fat | Fat | Neutral |
| Mammalian game meats | Salt & Fat | Fat | Salt & Fat |
| Beef, sheep & pork, unprocessed | Salt & Fat, Fat | Fat | Neutral |
| Poultry & feathered game | Salt & Fat, Fat | Fat | Neutral |
| Organ meats & offal, products & dishes | Salt & Fat | Fat | Salt & Fat |
| Processed meat | Salt & Fat | Salt & Fat, Fat | Salt & Fat |
| Fin fish | Salt & Fat | Fat | Salt & Fat |
| Crustacea & molluscs | Salt & Fat | Fat | Salt & Fat |
| Other sea & freshwater foods | Salt & Fat | Fat | Salt & Fat |
| Packed (commercially sterile) fish & seafood | Salt & Fat | Fat | Salt & Fat |
| Fish & seafood products | Salt & Fat, Fat | Fat | Salt & Fat |
| Mature legume & pulse, products & dishes | Neutral | Neutral, Fat | Neutral |
| Other fruiting vegetables | Neutral, Sweet, Salt & Fat, Fat | Neutral, Sweet, Fat | Neutral, Sweet, Sweet & Sour, Salt & Fat |

indicates a cluster that differed from clusters formed by the other two distance measures.

Several notable minor food groups and their clusters are presented in Table 6.5. For example, Manhattan distance clustered some meats as ‘fat’ only without ‘salt and fat’ and mature legume and pulse products or dishes as ‘fat’ instead of ‘neutral’. Maximum distance clustered cream and dairy milk as ‘neutral’ instead of ‘fat’.

Supplementary Table S2.5. Taste and mouthfeel values alongside percentage contribution to total energy intake from each taste cluster using Ward’s with Euclidean method, in (A) six, and (B) seven clusters.

| **A) 6 clusters** | | | | | | | | |
| --- | --- | --- | --- | --- | --- | --- | --- | --- |
|  | **Bitter**  (n=78) | **Neutral**  (n=834) | **Sweet**  (n=1007) | **Sweet Sour**  (n=357) | **Salt Fat**  (n=1456) | **Fat**  (n=814) |  |  |
| Energy (kcal) | 145.2 | 357.3 | 449.7 | 227.4 | 676.7 | 374.5 |  |  |
| Energy (%) | 7.0 | 17.3 | 21.8 | 11.0 | 32.8 | 18.1 |  |  |
| Sweet | 10.6±5.5 | 13.6±7.6 | 52.6±10.1 | 32.4±12.5 | 15.8±5.9 | 8.7±6.5 |  |  |
| Sour | 5.0±3.7 | 5.9±4.8 | 7.1±5.9 | 36.4±10.0 | 7.7±5.6 | 2.8±1.7 |  |  |
| Bitter | 55.4±4.4 | 7.8±6.7 | 3.3±2.6 | 6.4±6.5 | 2.5±2.2 | 2.4±3.2 |  |  |
| Umami | 0.7±0.3 | 3.1±1.9 | 0.7±0.4 | 2.2±2.7 | 17.0±6.0 | 8.0±5.6 |  |  |
| Salt | 1.6±0.8 | 10.4±7.0 | 9.0±4.4 | 9.3±10.3 | 34.6±9.0 | 13.6±6.4 |  |  |
| Fatty | 6.6±4.4 | 7.4±4.8 | 17.0±10.3 | 12.0±15.4 | 26.3±9.8 | 29.1±13.6 |  |  |
| **B) 7 clusters** | | | | | | | | |
|  | **Bitter**  (n=78) | **Neutral**  (n=834) | **Sweet**  (n=1007) | **Sweet Sour**  (n=357) | **Salt Fat**  (n=1456) | **Fat?**  (n=723) | **Fat 2?**  (n=91) |  |
| Energy (kcal) | 145.2 | 357.3 | 449.7 | 227.4 | 676.7 | 360.2 | 92.3 |  |
| Energy (%) | 7.0 | 17.1 | 21.6 | 10.9 | 32.5 | 17.3 | 4.4 |  |
| Sweet | 10.6±5.5 | 13.6±7.6 | 52.6±10.1 | 32.4±12.5 | 15.8±5.9 | 8.7±6.8 | 8.8±3.6 |  |
| Sour | 5.0±3.7 | 5.9±4.8 | 7.1±5.9 | 36.4±10.0 | 7.7±5.6 | 2.5±.4 | 4.5±2.6 |  |
| Bitter | 55.4±4.4 | 7.8±6.7 | 3.3±2.6 | 6.4±6.5 | 2.5±2.2 | 2.3±2.3 | 3.0±7.0 |  |
| Umami | 0.7±0.3 | 3.1±1.9 | 0.7±0.4 | 2.2±2.7 | 17.0±6.0 | 8.7±5.6 | 2.7±2.4 |  |
| Salt | 1.6±0.8 | 10.4±7.0 | 9.0±4.4 | 9.3±10.3 | 34.6±9.0 | 12.1±3.5 | 25.6±10.3 |  |
| Fatty | 6.6±4.4 | 7.4±4.8 | 17.0±10.3 | 12.0±15.4 | 26.3±9.8 | 24.6±4.5 | 65.0±6.7 |  |

To check if additional clusters would improve cluster formation, values were calculated again to compare between having six and seven clusters (Table 6.6). Five of the clusters remained the same. To form the seventh cluster, the sixth cluster was split into two clusters that were both highest in their fatty mouthfeel values (i.e., ‘Fat’ and ‘Fat 2?’). This did not lead to more meaningful and understandable clusters; hence, six clusters specification was chosen. Among the six clusters, ‘Salt and Fat’ cluster contributed the most to total energy intake, followed by ‘Sweet’, ‘Fat’, ‘Neutral’, ‘Sweet and Sour’ and lastly, ‘Bitter’ cluster contributed the least.

Supplementary Table S3.1. Standard for evaluating eating rate/duration categories.

| **No.** | **Food or Food Category** | **Category^1^** | **Code** |
| --- | --- | --- | --- |
| 1 | Bread  except bun, roll, muffin, untoasted white bread | hard | 4 |
| 2 | Croissant  e.g., chocolate croissant, croissant with ham and cheese | hard | 4 |
| 3 | Breakfast cereal  e.g., Kellogg’s special K | hard | 4 |
| 4 | Rice/oat/cereal porridge  e.g., rolled oats porridge | soft | 3 |
| 5 | Aged/ hard cheese ^2^  e.g., Gouda 48+, Emmental | hard | 4 |
| 6 | Fish  e.g., salmon; except fried | soft | 3 |
| 7 | Meat ^2^  e.g., pork fillet; except minced or processed (sausage etc.) | hard | 4 |
| 8 | Minced meat | soft | 3 |
| 9 | Processed meat  e.g., sausage; except deep fried | soft | 3 |
| 10 | Cooked meals  e.g., curry, wrap, hamburger, sandwich, mixed dishes | soft | 3 |
| 11 | Sushi  e.g., maki roll | soft | 3 |
| 12 | Egg  e.g., omelette | soft | 3 |
| 13 | Vegetables raw  e.g., rucola, spinach, lettuce, parsley | hard | 4 |
| 14 | Salad  e.g., Russian salad; except egg salad and Dutch ‘salad’ spreads | hard | 4 |
| 15 | Cooked/tinned vegetables  e.g., boiled swede, steamed broccoli, canned beans; except sprouts, corn | soft | 3 |
| 16 | Hard fruit  e.g., apple, pineapple, melon | hard | 4 |
| 17 | Soft fruit  e.g., strawberry, avocado | soft | 3 |
| 18 | Dried fruit  e.g., dried mango | hard | 4 |
| 19 | Fruit puree | semi | 2 |
| 20 | Potato/bean puree  e.g., mashed potato | soft | 3 |
| 21 | Relish  e.g., Piccalilli | soft | 3 |
| 22 | Compote  e.g. berry compote | soft | 3 |
| 23 | Preserve  e.g. marmalade | soft | 3 |
| 24 | Jam | semi | 2 |
| 25 | Jelly  e.g. fruit jelly | soft | 3 |
| 26 | Wine gum/ jelly ^2^  e.g. fruit gum | hard | 4 |
| 27 | Infant food puree  e.g. infant 6 months fruit puree | semi | 2 |
| 28 | Infant food sieved  e.g. infant sieved broccoli | soft | 3 |
| 29 | Paste  e.g. tahini | soft | 3 |
| 30 | Gravy  e.g. gravy powder prepared | semi | 2 |
| 31 | Dip  e.g. avocado dip | soft | 3 |
| 32 | Dressing  e.g. salad dressing | semi | 2 |
| 33 | Sauce  e.g. apple sauce, chili sauce | semi | 2 |
| 34 | Pasta sauce  e.g. ready-made Bolognese sauce in jar | soft | 3 |
| 35 | Pre-diluted syrup  e.g. pancake syrup, fruit syrup | semi | 2 |
| 36 | Diluted syrup | liquid | 1 |
| 37 | Custard  e.g. vanilla-chocolate duo custard | semi | 2 |
| 38 | Pudding  e.g. butterscotch pudding | soft | 3 |
| 39 | Mousse  e.g. chocolate mousse | soft | 3 |
| 40 | Powders  e.g. cocoa powder, curry powder | hard | 4 |
| 41 | Instant/pre-mix  e.g. herb mix, rice mix to add | hard | 4 |
| 42 | Soup without pieces  e.g. instant soup made from dry mix | semi | 2 |
| 43 | Soup with pieces  e.g. chicken soup with noodles and chicken | soft | 3 |
| 44 | Oil/ cooking spray  e.g. olive oil, sesame oil | semi | 2 |
| 45 | Butter/ margarine  e.g. unsalted butter, dairy blend | soft | 3 |
| 46 | Baked treats  e.g. cakes, eclairs, strudel, crumble, donut, pancake, waffle | soft | 3 |
| 47 | Puff pastry  e.g. pot pie with puff pastry, apple turnover | hard | 4 |
| 48 | Ice cream  e.g. Neapolitan | semi | 2 |
| 49 | Ice cream with nuts/ biscuits  e.g. cookies and cream ice-cream | soft | 3 |
| 50 | Thick drinks  e.g. smoothie, milkshake | liquid | 1 |
| 51 | Beer/ wine  e.g. rose, lager | liquid | 1 |
| 52 | Cocktail  e.g. gin and tonic, whisky sour | liquid | 1 |
| 53 | Hard alcohol ^2^  e.g. gin, whisky, liqueur | semi | 2 |

^1^Definitions as follows – liquid: foods that flow and do not require chewing before swallowing; semi-solid: foods that are predominantly processed by squeezing between tongue & palate; soft-solid: foods that require (initial) chewing between the molars but without crisp; hard-solid: crispy foods that require chewing between molars, generally producing sound. ^2^Categorised based on tested eating rates (van den Boer, Werts, et al., 2017), instead of category definition.

Differences between sexes in cluster contributions to food intake

Contribution to amount eaten (%)

|  | Male (n=3110) | Female (n=3305) |
| --- | --- | --- |
| Bitter | 25.3 | 18.7 |
| Neutral | 42.4 | 49.1 |
| Sweet | 13.2 | 12.2 |
| Sweet & Sour | 12.2 | 11.7 |
| Salt & Fat | 12 | 11.4 |
| Fat | 8.3 | 7.7 |

Supplementary Figure S3.1. Sex differences in percentage contributions of taste clusters to amount eaten.

Differences in taste amounts from the whole diet

|  | Male (n=3110) | Female (n=3305) |
| --- | --- | --- |
| Sweet | 53714 | 41258 |
| Sour | 28764 | 22086 |
| Bitter | 48820 | 35663 |
| Umami | 11096 | 8819 |
| Salt | 30083 | 24393 |
| Fat | 28633 | 23035 |

(B) Differences in contribution to total taste amount

Percentage contribution (%)

|  | Male (n=3110) | Female (n=3305) |
| --- | --- | --- |
| Sweet | 26.7088 | 26.5745 |
| Sour | 14.3026 | 14.2257 |
| Bitter | 24.2753 | 22.9707 |
| Umami | 5.51738 | 5.68037 |
| Salt | 14.9585 | 15.7117 |
| Fat | 14.2375 | 14.837 |

Supplementary Figure S3.2. Sex differences in (A) total taste amount and (B) percentage contributions to total taste amount.

(A) Differences in taste exposure from the diet

Taste exposure (score min)

|  | Male (n=3110) | Female (n=3305) |
| --- | --- | --- |
| Sweet | 94514 | 84155 |
| Sour | 39027 | 35078 |
| Bitter | 29096 | 24019 |
| Umami | 38485 | 29348 |
| Salt | 82696 | 64948 |
| Fat | 80280 | 62631 |

(B) Differences in contribution to total taste exposure

Percentage contribution (%)

|  | Male (n=3110) | Female (n=3305) |
| --- | --- | --- |
| Sweet | 23.9 | 25.4 |
| Sour | 11.4 | 11.6 |
| Bitter | 15.1 | 13.4 |
| Umami | 9.7 | 9.5 |
| Salt | 21.1 | 21.5 |
| Fat | 18.9 | 18.6 |

Supplementary Figure S3.3. Sex differences in (A) taste exposure and (B) percentage contributions to total taste exposure.

Supplementary Table S4.1. Percentage (and number) of foods in each texture category across the countries.

| N (%) | **FR** | **NL** | **UK** | **AU** | **US** |
| --- | --- | --- | --- | --- | --- |
| **Liquid** | 9.9 *(1706)* | 16.9 *(314)* | 11.0 *(304)* | 10.5 *(479)* | 9.1 *(440)* |
| **Semi-solid** | 14.7 *(2531)* | 12.9 *(241)* | 10.2 *(282)* | 7.3 *(334)* | 6.6 *(321)* |
| **Soft-solid** | 48.1 *(8299)* | 36.1 *(673)* | 41.7 *(1154)* | 45.4 *(2062)* | 52.7 *(2554)* |
| **Hard-solid** | 27.4 *(4725)* | 34.1 *(635)* | 37.1 *(1025)* | 36.7 *(1670)* | 31.6 *(1535)* |

FR: France. NL: the Netherlands. UK: the United Kingdom. AU: Australia. US: the United States.

All five countries had the largest proportion of their consumed foods assigned to the soft-solid category (36.1-52.7%) and the least number of their consumed foods assigned to the semi-solid category, except for France, whose diet had the least number of consumed foods assigned to the liquid category.

Supplementary Table S4.2. Minor food groups from each country in each cluster.

The French diet resulted in six clusters where its ‘Sweet and Fat’ and ‘Sour and Fat’ clusters were unique compared to other countries. Its ‘Neutral’ cluster was the highest in energy contribution (48.7%), followed by ‘Bitter’ (16.1%), ‘Sweet’ (15.4%), ‘Umami, Salt and Fat’ (10.9%), ‘Sweet and Fat’ (6.5%) and lastly, ‘Sour and Fat’ cluster (4.5%). The Dutch diet also consisted of six clusters – ‘Neutral’ (39.4%), ‘Bitter’ (26.6%), ‘Sweet and Sour’ (17.1%), ‘Sweet’ (10.0%), ‘Umami, Salt and Fat’ (5.7%) and ‘Salt and Fat’ cluster (2.9%). The population diet from the UK resulted in six clusters, with ‘Neutral’ (37.3%) contributing the most to total energy intake, then ‘Bitter’ (28.2%), ‘Sweet’ (16.0%), ‘Sweet and Sour’ (13.0%), ‘Fat’ (6.1%), and ‘Salt and Fat’ cluster (5.9%). The population diet from Australia resulted in six clusters similar to that from Australia – ‘Neutral’ (45.7%), ‘Bitter’ (22.2%), ‘Sweet’ (12.7%), ‘Sweet and Sour’ (11.9%), ‘Salt and Fat’ (11.7%) and lastly, ‘Fat’ cluster (8.0%). The population diet from the US was the only one that consisted of seven clusters. Its ‘Neutral’ cluster contributed the most to total energy intake (44.1%), followed by ‘Bitter’ (20.1%), ‘Sweet’ (14.5%), ‘Sweet and Sour’ (12.1%), ‘Salt and Fat’ (11.7%), ‘Fat’ (5.1%) and lastly, ‘Umami, Salt and Fat’ cluster (4.4%).

| **Cluster: Neutral** | |
| --- | --- |
| **FR** | Cereals and cereal primary derivatives; Breakfast cereals; Starches; Pasta, doughs and similar products; Starchy roots and tubers; Dishes, including ready-to-eat meals; Processed or preserved vegetables and similar; Soups and salads; Leafy vegetables; Herbs and edible flowers; Stems/stalks eaten as vegetables; Algae and prokaryote organisms; Fruiting vegetables; Legumes with pod; Fruit used as fruit; Root and tuber vegetables (excluding starchy- and sugar-); Flowering brassica; Fungi, mosses and lichens; Legumes; Processed legumes, nuts, oilseeds and spices; Bulb vegetables; Sprouts, shoots and similar; Spices; Nuts, oilseeds and oilfruits; Meat and dairy imitates; Amphibians, reptiles, snails, insects; Processed eggs; Unprocessed eggs; Drinking water; Unsweetened spirits and liqueurs; Milk, whey and cream; Milk and dairy powders and concentrates; Cheese; Bread and similar products; Fine bakery wares; Mammals and birds meat; Animal meat dried; Marinated meat; Canned-tinned meat; Fish; Fish and seafood processed; Molluscs; Hot drinks and similar (coffee, cocoa, tea and herbal drinks); Condiments; Fried or extruded cereal or root-based products |
| **NL** | Cereals and cereal products; Eggs; Meat and poultry; Fish; Legumes; Nuts and seeds; Bread; Milk and milk products; Meat substitutes and dairy substitutes; Vegetables; Non-alcoholic beverages; Savoury bread spreads; Pastry and biscuits; Fruits; Herbs and spices; Potatoes and tubers; Cheese; Cold meat cuts; Savoury sauces. |
| **UK** | Other cereals; Other rice incl homemade dishes; Beans & pulses incl ready meal & homemade dishes; Other pasta incl homemade dishes; Brown granary & wheatgerm bread; Other bread; White bread; Wholemeal bread; Other breakfast cereals; High fibre breakfast cereals; Biscuits mfg; Other eggs & egg dishes incl homemade; Skimmed milk; Other milk; Other potatoes incl homemade dishes; Other chicken incl homemade recipe dishes; Other white fish incl homemade dishes; Other shellfish incl homemade dishes; Other vegetables incl homemade dishes; Green beans not raw; Meat alternatives incl ready meals & homemade dish; Salad & other raw vegetables; Leafy green vegetables not raw; Peas not raw; Other fried / roast potatoes incl homemade dishes; Other potato products & dishes mfg; Tomatoes not raw; Nuts & seeds; Savoury sauces pickles gravies & condiments; Mfg oily fish products incl ready meals; Other canned tuna incl homemade dishes; Tap water only; Herbal tea; Chocolate confectionery; Bottled water still or carbonated; Soft drinks not low calorie carbonated; Pasta products & ready meals; Other fruit not canned; Yogurt |
| **AU** | Tea; Waters, municipal and bottled, unflavoured; Flours and other cereal grains and starches; Regular breads, and bread rolls (plain/unfilled/untopped varieties); English-style muffins, flat breads, and savoury and sweet breads; Dry soup mix; Seeds and seed products; Gravies and savoury sauces; Cabbage, cauliflower and similar brassica vegetables; Carrot and similar root vegetables; Leaf and stalk vegetables; Peas and beans; Tomato and tomato products; Other fruiting vegetables; Other vegetables and vegetable combinations; Dishes where vegetable is the major component; Mature legumes and pulses; Mature legume and pulse products and dishes; Spirits; Other alcoholic beverages; Yeast, and yeast vegetable or meat extracts; Herbs, spices, seasonings and stock cubes; Essences; Chemical raising agents and cooking ingredients; Infant cereal products; Breakfast cereals, hot porridge style; Breakfast cereals, ready to eat; Cheese; Dips; Dried fruit, preserved fruit; Eggs; Fin fish (excluding commercially sterile); Mixed dishes where cereal is the major ingredient; Muesli or cereal style bars; Nuts and nut products; Packed (commercially sterile) fish and seafood; Pasta and pasta products (without sauce); Pome fruit; Potatoes; Poultry and feathered game; Soup, homemade from basic ingredients |
| **US** | Milk, nonfat; Milk substitutes; Chicken, whole pieces; Fish; Shellfish; Vegetable mixed dishes; Poultry mixed dishes; Eggs & omelets; Beans, peas, legumes; Processed soy products; Pasta, noodles, cooked grains; Nuts & seeds; Yeast breads; Bagels & English muffins; Rolls & buns; Biscuits, muffins, quick breads; Tortillas; Cookies & brownies; Crackers, excludes saltines; Grits & other cooked cereals; Oatmeal; Rice; Ready-to-eat cereal, lower sugar; Other sandwiches; Soups; White potatoes, baked or boiled; Mashed potatoes & white potato mixtures; Dark green vegetables, excludes lettuce; Lettuce & lettuce salads; Other vegetables & combi; Other red & orange vegetables; Tomato-based condiments; String beans; Onions; Mustard & other condiments; Coffee; Tea; Tap water; Bottled water |
| **Cluster: Bitter** | |
| **FR** | Confectionery including chocolate; Hot drinks and similar (coffee, cocoa, tea and herbal drinks); Mixed alcoholic drinks; Beer and beer-like beverage; Leafy vegetables; Animal and vegetable fats/oils |
| **NL** | Non-alcoholic beverages (including coffee, tea); Alcoholic beverages; Vegetables; Fruits; Nuts and seeds; Milk and milk products; Herbs and spices; Pastry and biscuits |
| **UK** | Coffee; Tea; Beers & lagers; Low alcohol & alcohol free beer & lager; Salad & other raw vegetables |
| **AU** | Coffee & coffee substitutes; Beers; Chocolate & chocolate-based confectionery; Leaf & stalk vegetables; Tea |
| **US** | Dark green vegetables; Lettuce & salads; Mustard & other condiments; Red, orange & other vegetables; Salad dressings; Coffee; Tea; Flavored or carbonated water; Beer; Liquor & cocktails |
| **Cluster: Sweet** | |
| **FR** | Sugar plants; Liquid or gel separated from plant RPCs; Sugar and other sweetening ingredients; Beverage concentrates; Fruit/vegetable juices and nectars; Concentrated or dehydrated fruit/vegetables juices; Water-based beverages, licorice flavour; Drinking water; Mixed alcoholic drinks; Soups and salads; Fruit; Root and tuber vegetables; Starchy roots and tubers; Dishes, including ready-to-eat meals; Processed or preserved vegetables and similar; Legumes; Fruiting vegetables; Stems/stalks eaten as vegetables; Herbs and edible flowers; Nuts, oilseeds and oilfruits; Processed fruit products; Meat and dairy imitates; Fermented milk or cream; Cheese; Dairy dessert and similar; Breakfast cereals; Fine bakery wares; Processed legumes, nuts, oilseeds and spices; Confectionery including chocolate; Water-based beverages; Beer and beer-like beverage; Wine and wine-like drinks; Unsweetened spirits and liqueurs; Water-based sweet desserts; Bread and similar products; Condiments |
| **NL** | Fruits; Nuts and seeds; Cereals and cereal products; Pastry and biscuits; Bread; Milk and milk products; Sugar, sweets and sweet sauces; Non-alcoholic beverages; Miscellaneous foods; Meat substitutes and dairy substitutes; Legumes; Vegetables |
| **UK** | Cereal based milk puddings homemade; Buns cakes & pastries mfg; White bread (not high fibre not multiseed bread); High fibre breakfast cereals; Other breakfast cereals (not high fibre); Biscuits mfg / retail; Biscuits homemade; Buns cakes & pastries homemade; mfg egg products incl ready meals; Other cereal based puddings homemade; Other cereal based puddings mfg; Fruit pies mfg; Fruit pies homemade; Sponge puddings homemade; Cereal based milk puddings mfg; Ice cream; Whole milk; Semi skimmed milk; Other milk; Cream; Salad & other raw vegetables; Carrots raw; Carrots not raw; Peas not raw; Other vegetables incl homemade dishes; Apples & pears not canned; Other fruit not canned; Canned fruit in syrup; Canned fruit in juice; Bananas; Citrus fruit not canned; Nuts & seeds;Sugar; Sweet spreads fillings & icing; Preserves; Chocolate confectionery; Sugar confectionery; Beverages dry weight; Fruit juice; Soft drinks not low calorie carbonated; Dairy desserts homemade; Fromage frais & dairy desserts mfg; Sponge puddings mfg; Soft drinks low calorie carbonated; Soft drinks not low calorie conc; Bottled water still or carbonated; Yogurt; Soft drinks not low cal rtd still |
| **AU** | Soft drinks, and flavoured mineral waters; Other beverage flavourings and prepared beverages; Breakfast cereals, ready to eat; Sweet biscuits; Cakes, muffins, scones, cake-type desserts; Batter-based products; Pome fruit; Tropical and subtropical fruit; Mixtures of two or more groups of fruit; Dried fruit, preserved fruit; Mixed dishes where fruit is the major component; Frozen milk products; Custards; Other dishes where milk or a milk product is the major component; Flavoured milks and milkshakes; Dairy milk substitutes, flavoured; Soy-based ice confection; Sugar, honey and syrups; Jam and lemon spreads, chocolate spreads, sauces; Dishes and products other than confectionery where sugar is the major component; Chocolate and chocolate-based confectionery; Fruit, nut and seed-bars; Muesli or cereal style bars; Other confectionery; Formula dietary foods; Infant foods; Breakfast cereals, hot porridge style; Carrot and similar root vegetables; Citrus fruit; Cordials; Dairy milk (cow, sheep and goat); Dairy milk substitutes, unflavoured; English-style muffins, flat breads, and savoury and sweet breads; Fruit and vegetable juices, and drinks; Other fruit; Other fruiting vegetables; Pastries; Stone fruit |
| **US** | Flavored milk, whole; Milk substitutes; Yogurt, regular; Ice cream & frozen dairy desserts; Flavored milk, nonfat; Flavored milk, reduced fat; Flavored milk, lowfat; Milk shakes & other dairy drinks; Smoothies & grain drinks; Cream cheese, sour cream, whipped cream; Pudding; Cakes & pies; Nuts & seeds; Yeast breads; Doughnuts, sweet rolls, pastries; Bagels & English muffins; Biscuits, muffins, quick breads; Cookies & brownies; Pancakes, waffles, French toast; Cereal bars; Oatmeal; Ready-to-eat cereal, higher sugar; Ready-to-eat cereal, lower sugar; Dried fruits; Apples; Bananas; Melons; Other fruits & fruit salads; Peaches & nectarines; Berries; Gelatins, ices, sorbets; Other starchy vegetables; Carrots; Other vegetables & combi; Other red & orange vegetables; Tomatoes; Corn; Vegetable mixed dishes; Soups; Other fruit juice; Sugars & honey; Jams, syrups, toppings; Candy not containing chocolate; Candy containing chocolate; Coffee; Soft drinks; Diet soft drinks; Fruit drinks; Other diet drinks; Enhanced or fortified water; Nutritional beverages |
| **Cluster: Sweet and Sour** | |
| **FR** | - |
| **NL** | Fruits; Vegetables; Milk and milk products; Non-alcoholic beverages; Alcoholic beverages; Sugar, sweets and sweet sauces; Cereals and cereal products; Pastry and biscuits; Miscellaneous foods; Meat substitutes and dairy substitutes. |
| **UK** | Buns, cakes & pastries homemade; Other milk; Cream; Other cheese; Cottage cheese; Yogurt; Salad & other raw vegetables; Beans & pulses incl ready meal & homemade dishes; Other fruit not canned; Fruit juice; Canned fruit in juice; Sugar confectionery; Preserves; Soft drinks not low calorie conc; Soft drinks low calorie conc; Soft drinks not low calorie still; Soft drinks low calorie still; Cider & perry; Wine; Fortified wine; Savoury sauces pickles gravies & condiments; Other vegetables incl homemade dishes; Alcoholic soft drinks; Smoothies 100% fruit &/or juice; Soft drinks not low calorie carbonated; Beers & lagers; Low alcohol & alcohol free wine; Tomatoes raw |
| **AU** | Fruit and vegetable juices, and drinks; Cordials; Electrolyte, energy and fortified drinks; Tea; Berry fruit; Citrus fruit; Stone fruit; Other fruit; Yoghurt; Cream; Soy-based yoghurts; Pickles, chutneys and relishes; Salad dressings; Beers; Wines; Cider and perry; Infant drinks; Cabbage, cauliflower and similar brassica vegetables; Cakes, muffins, scones, cake-type desserts; Carrot and similar root vegetables; Cheese; Custards; Dips; Dried fruit, preserved fruit; Frozen milk products; Mixed dishes where cereal is the major ingredient; Mixtures of two or more groups of fruit; Other beverage flavourings and prepared beverages; Other confectionery; Pastries; Pome fruit; Soft drinks, and flavoured mineral waters; Tomato and tomato products; Tropical and subtropical fruit. |
| **US** | Milk, reduced fat; Yogurt, regular; Yogurt, Greek; Dips, gravies, other sauces; Ice cream & frozen dairy desserts; Cream cheese, sour cream, whipped cream; Cottage/ricotta cheese; Cheese sandwiches; Other sandwiches; Egg/breakfast sandwiches; Mustard & other condiments; Peanut butter & jelly sandwiches; Fruit drinks; Cakes & pies; Ready-to-eat cereal, lower sugar; Turnovers & other grain-based items; Citrus fruits; Citrus juice; Other fruit juice; Dried fruits; Other fruits & fruit salads; Apples; Grapes; Berries; Apple juice; Vegetable juice; Tomatoes; Olives, pickles, pickled vegetables; Tomato-based condiments; Mayonnaise; Salad dressings & vegetable oils; Tea; Soft drinks; Beer; Wine; Sport & energy drinks; Diet sport & energy drinks |
| **Cluster: Salt and Fat** | |
| **FR** | - |
| **NL** | Cheese; Fats and oils; Savoury sauces; Savoury bread spreads; Meat substitutes and dairy substitutes; Mixed dishes; Savoury snacks; Vegetables; Potatoes and tubers. |
| **UK** | Other pasta incl homemade dishes; Rice mfg products & ready meals; Other cereals; Biscuits mfg / retail; Crisps & savoury snacks; Other cheese; Cheddar cheese; Other bacon & ham incl homemade dishes; Other pork incl homemade recipe dishes; Mfg coated chicken / turkey products; Mfg chicken products incl ready meals; Homemade meat pies & pastries; Mfg meat pies & pastries; Other meat incl homemade recipe dishes; Other sausages incl homemade dishes; Other meat products mfg incl ready meals; Baked beans; Other rice incl homemade dishes; Mfg beef products incl ready meals; Other oily fish incl homemade dishes; Mfg oily fish products incl ready meals; Mfg canned tuna products incl ready meals; Other shellfish incl homemade dishes; Other fruit not canned; Nuts & seeds; Fruit juice; Savoury sauces pickles gravies & condiments; Soup mfg/ retail; Soup homemade; Pasta mfg products & ready meals; Other chicken / turkey incl homemade recipe dishes; Other beef & veal incl homemade recipe dishes; White bread (not high fibre not multiseed bread); Other mfg vegetable product; Mfg egg products incl ready meals; Mfg white fish products incl ready meals; Meat alternatives incl ready meals & homemade dish; Reduced fat spread (not pufa); Ready meals based on sausages; Soft drinks not low calorie rtd still |
| **AU** | Pasta & pasta products (without sauce); Savoury biscuits; Pastries; Mixed dishes where cereal is the major ingredient; Unspecified fats; Crustacea & molluscs; Other sea & freshwater foods; Packed fish & seafood; Fish & seafood products; Mixed dishes with fish or seafood as the major component; Mammalian game meats; Organ meats & offal, products & dishes; Processed meat; Mixed dishes where beef, sheep, pork or mammalian game is the major component; Mixed dishes where sausage, bacon, ham or other processed meat is the major component; Mixed dishes where poultry or feathered game is the major component; Cheese; Meat substitutes; Dishes where meat substitutes are the major component; Soup, homemade from basic ingredients or prepared from dry soup mix or prepared from condensed or sold ready to eat; Canned condensed soup; Gravies & savoury sauces; Dips; Potato or corn snacks; Extruded or reformed snacks; Other snacks; Yeast, & vegetable or meat extracts; Beef, sheep & pork, unprocessed; Cheese substitute; Dishes where vegetable is the major component; English-style muffins, flat breads, & savoury & sweet breads; Fin fish; Flours & other cereal grains & starches; Herbs, spices, seasonings and stock cubes; Mature legume & pulse products & dishes; Nuts & nut products; Other fruiting vegetables; Other vegetables & vegetable combi; Pickles, chutneys & relishes; Potatoes; Poultry & feathered game; Regular breads, & bread rolls; Salad dressings; Stuffings; Tomato products |
| **US** | Dips, gravies, other sauces; Cheese; Cottage/ricotta cheese; Pizza; Soups; Beef, excludes ground; Ground beef; Pork; Cold cuts & cured meats; Lamb, goat, game; Chicken, whole pieces; Turkey, duck, other poultry; Liver & organ meats; Fish; Shellfish; Stir-fry & soy-based sauce mixtures; Poultry mixed dishes; Pasta sauces, tomato-based; Meat mixed dishes; Rice mixed dishes; Burritos & tacos; Burger; Chicken/turkey sandwiches; Other sandwiches; Frankfurter sandwiches; Beans, peas, legumes; Processed soy products; Nuts & seeds; Rolls & buns; Yeast breads; Crackers, excludes saltines; Turnovers & other grain-based items; Doughnuts, sweet rolls, pastries; Saltine crackers; Tortilla, corn, other chips; Popcorn; Pasta, noodles, cooked grains; Other Mexican mixed dishes; Nachos; Pasta mixed dishes, excludes macaroni & cheese; Macaroni & cheese; Egg rolls, dumplings, sushi; Other vegetables & combi; Potato chips; Mashed potatoes potato mixtures; French fries & other fried potatoes; Lettuce & salads; Corn; Vegetable mixed dishes; Mayonnaise |
| **Cluster: Fat** | |
| **FR** | - |
| **NL** | - |
| **UK** | Pasta mfg products & ready meals; White bread (not high fibre not multiseed bread); Cream; Other cheese; Butter; Other cooking fats & oils not pufa; Block margarine; Pufa oils; Other beef & veal incl homemade recipe dishes; Other lamb incl homemade recipe dishes; Other chicken / turkey incl homemade recipe dishes; Liver & dishes; Mfg beef products incl ready meals; Burgers & kebabs purchased; Meat alternatives incl ready meals & homemade dish; White fish coated or fried; Mfg shellfish products incl ready meals; Other vegetables incl homemade dishes; mfg vegetable products incl cream; Chips purchased; Other mfg potato products fried/ baked; Nuts & seeds; Pizza; Pufa low fat spread; Buns cakes & pastries mfg; Mfg lamb products incl ready meals; Reduced fat spread; Rice mfg products & ready meals; Mfg pork products incl ready meals; Other white fish incl homemade dishes; Mfg coated chicken products incl ready meals; Other pork incl homemade dishes; Low fat spread not pufa; Ready meals based on bacon & ham; Other cereals |
| **AU** | Butters; Dairy blends; Margarine & table spreads; Plant oils; Other fats; Eggs; Dishes where egg is the major ingredient; Beef, sheep & pork, unprocessed; Poultry & feathered game; Dairy milk; Dairy milk substitutes, unflavoured; Nuts & nut products; Potatoes; Batter-based products; Cabbage, cauliflower & similar brassica vegetables; Carrot & similar root vegetables; Cheese; Corn snacks; Cream; Crustacea & molluscs; Dips; Dishes where vegetable is the major component; Fish & seafood products (homemade & takeaway); Flavoured milks & milkshakes; Leaf & stalk vegetables; Meat substitutes; Mixed dishes where cereal is the major ingredient; Other fruiting vegetables; Other vegetables & vegetable combi; Pasta & pasta products (without sauce); Pastries; Seeds & seed products |
| **US** | Milk, reduced fat; Milk, whole; Milk, lowfat; Milk substitutes; Cream & cream substitutes; Ice cream & frozen dairy desserts; Cottage/ricotta cheese; Beef, excludes ground; Butter & animal fats; Chicken, whole pieces; Chicken patties, nuggets & tenders; Fish; Dips, gravies, other sauces; Processed soy products; Nuts & seeds; Nutrition bars; Turnovers & other grain-based items; Pasta mixed dishes; French fries & other fried white potatoes; White potatoes, baked or boiled; Mashed potatoes & white potato mixtures; Carrots; Tomatoes; Vegetable mixed dishes; Onions; Margarine; Salad dressings & vegetable oils; Candy containing chocolate |
| **Cluster: Umami, Salt and Fat** | |
| **FR** | Mammals and birds meat; Animal liver; Animal kidney; Animal other organs (edible offals non-muscle); Animal other slaughtering products; Canned-tinned meat; Marinated meat; Meat and dairy imitates; Fat emulsions and blended fats; Breakfast cereals; Soups and salads; Fine bakery wares; Cheese; Bread and similar products; Animal meat dried; Processed whole meat products; Preserved fat tissues; Sausages; Meat specialties; Fish; Fish and seafood processed; Molluscs; Crustaceans; Fish offal; Fruit/vegetable juices and nectars; Mixed and other not listed condiments; Savoury extracts and sauce ingredients; Fried or extruded cereal or root-based products; Dishes, including ready-to-eat meals; Condiments; Processed or preserved vegetables and similar; Spices; Nuts, oilseeds and oilfruits; Processed fruit products; Flowers used as vegetables; Pasta, doughs and similar products |
| **NL** | Meat and poultry; Fish; Savoury snacks; Legumes; Bread; Cold meat cuts; Savoury sauces; Herbs and spices; Miscellaneous foods; Cheese; Meat substitutes and dairy substitutes; Potatoes and tubers; Mixed dishes; Savoury bread spreads; Soups; Nuts and seeds; Cereals and cereal products; Vegetables. |
| **UK** | - |
| **AU** | - |
| **US** | Dips, gravies, other sauces; Cheese; Cream cheese, sour cream, whipped cream; Cold cuts & cured meats; Ground beef; Bacon; Chicken, whole pieces; Chicken patties, nuggets & tenders; Frankfurters; Sausages; Fish; Shellfish; Meat mixed dishes; Pasta sauces, tomato-based; Poultry mixed dishes; Seafood mixed dishes; Fried rice & lo/chow mein; Soups; Tortilla, corn, other chips; Soy-based condiments; Processed soy products; Nuts & seeds; Yeast breads; Turnovers & other grain-based items; rackers, excludes saltines; Pretzels/snack mix; Egg rolls, dumplings, sushi; Pasta mixed dishes, excludes macaroni & cheese; Potato chips; Vegetable juice; Mustard & other condiments; Olives, pickles, pickled vegetables |
| **Cluster: Sweet and Fat** | |
| **FR** | Fruit; Root and tuber vegetables (excluding starchy- and sugar-); Bulb vegetables; Nuts, oilseeds and oilfruits; Extracts of plant origin; Water-based beverages; Hot drinks and similar (coffee, cocoa, tea and herbal drinks); Meat and dairy imitates; Pasta, doughs and similar products; Processed fruit products; Dairy dessert and similar; Dishes, including ready-to-eat meals; Confectionery including chocolate; Soups and salads; Milk, whey and cream; Milk and dairy powders and concentrates; Cheese; Fine bakery wares; Water-based sweet desserts; Sugar and other sweetening ingredients (excluding intensive sweeteners); Dessert sauces/toppings; Coffee, cocoa, tea and herbal ingredients; Bread and similar products; Cereals and cereal primary derivatives; Breakfast cereals; Fruit/vegetable juices and nectars |
| **NL** | - |
| **UK** | - |
| **AU** | - |
| **US** | - |
| **Cluster: Sour and Fat** | |
| **FR** | Fermented milk or cream; Cheese; Dairy dessert and similar; Fats and oils and primary derivatives thereof; Animal and vegetable fats/oils; Fat emulsions and blended fats; Preserved fat tissues; Soups and salads; Milk, whey and cream; Fine bakery wares; Fruit/vegetable juices and nectars; Condiments; Dishes, including ready-to-eat meals (excluding soups and salads); Processed or preserved vegetables and similar; Processed legumes, nuts, oilseeds and spices; Meat and dairy imitates |
| **NL** | - |
| **UK** | - |
| **AU** | - |
| **US** | - |

AU: Australia. FR: France. NL: the Netherlands. UK: the United Kingdom. US: the United States. Mfg.: manufactured. Incl.: including.

Note: cluster analyses were undertaken on individual foods. Where minor food groups contained foods with differing taste profiles, it is possible that the minor food group is represented in more than one taste cluster for any one country.

Supplementary Table S4.3. Contribution of food intake (grams) per taste across the countries calculated by summing up percentages of clusters in which the taste appears in.

| **(%)** | **FR** | **NL** | **UK** | **AU** | **US** |
| --- | --- | --- | --- | --- | --- |
| **Sweet** | 29.1  *(3.3+15.4)* | 18.6  *(8.6+10.0)* | 22.5  *(6.5+16.0)* | 18.7  *(12.7+6.0)* | 20.6  *(14.5+6.1)* |
| **Sour** | 2.3 | 8.6 | 6.5 | 6.0 | 6.1 |
| **Bitter** | 16.1 | 26.6 | 28.2 | 22.2 | 20.1 |
| **Umami** | 3.6 | 1.9 | - | - | 1.5 |
| **Salt** | 3.6 | 3.4  *(1.9+1.5)* | 3.0 | 5.9 | 7.4  *(1.5+5.9)* |
| **Fat** | 9.2  *(3.6+3.3+2.3)* | 3.4  *(1.9+1.5)* | 9.1  *(6.1+3.0)* | 13.9  *(8.0+5.9)* | 12.5  *(1.5+5.1+5.9)* |

FR: France. NL: the Netherlands. UK: the United Kingdom. AU: Australia. US: the United States. Note: Neutral was excluded as it was considered as neutral-tasting and did not contribute to any of the taste and mouthfeel.

Supplementary Table S4.4. Estimated marginal means^1^ of absolute and percentage contributions of dietary taste exposure from nationally representative diets of France (FR), the Netherlands (NL), the United Kingdom (UK), Australia (AU) and the United States (US).

| **US** | 81341 ± 473  (24.9 ± 0.095) | 32843 ± 256  (10.3 ± 0.060) | 22757 ± 174  (7.31 ± 0.057) | 33647 ± 198  (11.1 ± 0.052) | 73773 ± 302  (24.1 ± 0.068) | 67747 ± 252  (21.9 ± 0.063) | 322083 ± 1128 | 2986 ± 16.0 | 2013 ± 10.1 | ^1^ These values are estimated marginal means ± SE after removing outliers from each analysis. |
| --- | --- | --- | --- | --- | --- | --- | --- | --- | --- | --- |
| **AU** | 89051 ± 374  (26.4 ± 0.076) | 36605 ± 203  (11.0 ± 0.047) | 25825 ± 138  (8.09 ± 0.045) | 31408 ± 155  (9.99 ±0.041) | 70512 ± 237  (22.3 ± 0.054) | 69139 ± 199  (21.7 ± 0.050) | 331422 ± 892 | 3074 ± 12.6 | 1982 ± 7.9 |  |
| **UK** | 82782 ± 909  (25.9 ± 0.182) | 35439 ± 492  (11.1 ± 0.115) | 29110 ± 335  (9.44 ± 0.110) | 28497 ± 369  (9.28 ± 0.099) | 65960 ± 567  (21.4 ± 0.131) | 69588 ± 477  (22.8 ± 0.121) | 317581 ± 2165 | 2722 ± 30.1 | 1829 ± 19.1 |  |
| **NL** | 80160 ± 777  (26.5 ± 0.155) | 36803 ± 421  (12.1 ±0.098) | 34767 ± 286  (11.6 ±0.094) | 20694 ± 316  (7.15 ± 0.085) | 59407 ± 487  (20.5 ± 0.112) | 63987 ± 410  (22.0 ± 0.103) | 297972 ± 1851 | 2942 ± 25.6 | 2140 ± 16.5 |  |
| **FR** | 88422 ± 753  (25.5 ± 0.151) | 46849 ± 408  (13.5 ± 0.095) | 29875 ± 277  (8.90 ± 0.091) | 30930 ± 310  (9.28 ±0.082) | 68704 ± 474  (20.7 ± 0.109) | 72465 ± 399  (22.0 ± 0.100) | 342573 ± 1795 | 3010 ± 24.9 | 2073 ± 16.0 |  |
| **score min**  **(%)** | **Sweet** | **Sour** | **Bitter** | **Umami** | **Salt** | **Fat** | **Total** | **Consumed quantity per day, g** | **Energy intake per day, kcal** |  |

Figure S4.1. Taste clusters, their average intake amounts (g) and percentage contribution (%) to the diets of France (FR) using the FR taste database, the Netherlands (NL) using the NL taste database, the United Kingdom (UK) using the Australian (AU) database, AU using the AU taste database, and the United States (US) using the AU database.

Figure S4.2. Stacked plots of each taste and mouthfeel in (A) absolute values and (B) percentage contribution to total dietary taste exposure for France (FR) using the FR taste database, the Netherlands (NL) using the NL taste database, the United Kingdom (UK) using the AU database, Australia (AU) using the AU taste database and the United States (US) using the AU database. Bars labelled with different letters are different at *p* < .001, using Bonferroni-corrected comparisons to compare total taste exposure, where ‘a’ always represents the smallest value.
